# The Arrival of *Homo sapiens* into the Southern Cone at 14,000 Years Ago

**DOI:** 10.1371/journal.pone.0162870

**Published:** 2016-09-28

**Authors:** Gustavo G. Politis, María A. Gutiérrez, Daniel J. Rafuse, Adriana Blasi

**Affiliations:** 1 Instituto de Investigaciones Arqueológicas y Paleontológicas del Cuaternario Pampeano (INCUAPA-CONICET), Facultad de Ciencias Sociales, Universidad Nacional del Centro de la Provincia de Buenos Aires, Olavarría, Buenos Aires, Argentina; 2 Comisión de Investigaciones Científica de la Provincia de Buenos Aires (CIC), Museo de Ciencias Naturales, Universidad Nacional de La Plata, La Plata, Buenos Aires, Argentina; Max Planck Institute for the Science of Human History, GERMANY

## Abstract

The Arroyo Seco 2 site contains a rich archaeological record, exceptional for South America, to explain the expansion of *Homo sapiens* into the Americas and their interaction with extinct Pleistocene mammals. The following paper provides a detailed overview of material remains found in the earliest cultural episodes at this multi-component site, dated between ca. 12,170 ^14^C yrs B.P. (ca. 14,064 cal yrs B.P.) and 11,180 ^14^C yrs B.P. (ca. 13,068 cal yrs B.P.). Evidence of early occupations includes the presence of lithic tools, a concentration of Pleistocene species remains, human-induced fractured animal bones, and a selection of skeletal parts of extinct fauna. The occurrence of hunter-gatherers in the Southern Cone at ca. 14,000 cal yrs B.P. is added to the growing list of American sites that indicate a human occupation earlier than the Clovis dispersal episode, but posterior to the onset of the deglaciation of the Last Glacial Maximum (LGM) in the North America.

## Introduction

The current data from southern South America suggests *Homo sapiens* expanded into the Americas during a period earlier than the Clovis hunters of North America (older than ca. 11,500 ^14^C yrs B.P.) [1]. Currently, Monte Verde II is considered by most archaeologists as a prime example of this pre-Clovis occupation [2,3]. New data from the Pampas region of Argentina, support the association of extinct Pleistocene fauna and cultural remains at the Arroyo Seco 2 site (AS2), dated to 12,170 ^14^C yrs B.P. (13,814–14,147 cal yrs B.P.) [4].

**Fig 1:**
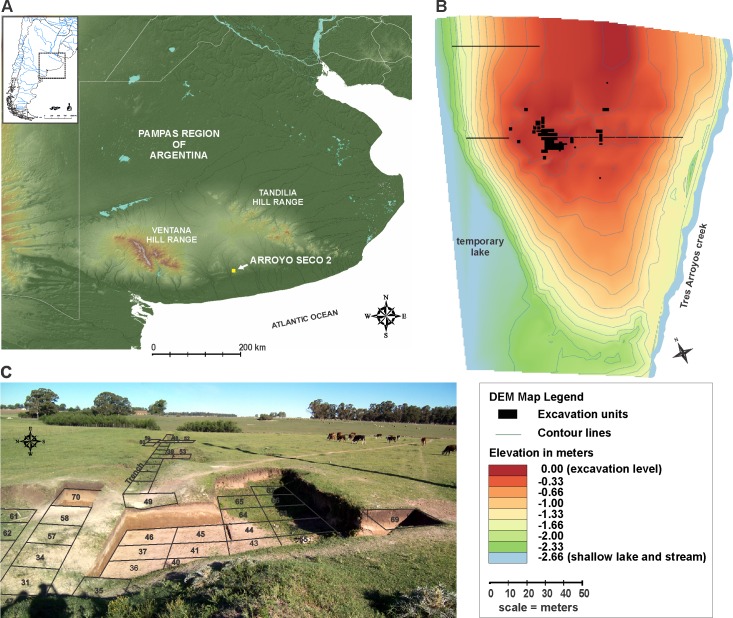
The Arroyo Seco 2 site. (A) Geographic location of the AS2 site. (B) Digital Elevation Model (DEM) of the knoll and location of the excavation units (S1 Fig). (C) Photograph of central excavation units and trench.

The AS2 site is located just outside the city of Tres Arroyos, in the Pampa region of Argentina. It is an open-air archaeological site situated on a low lying knoll between a small temporary lake and a shallow creek (38°21'38" S, 60°14'39" W) (Fig 1 and S1 File). The AS2 site is one of three archaeological sites in the locality, and was discovered and test pitted by archaeology amateurs from Tres Arroyos in the early 1970s. The first systematic fieldwork began in 1977, including the excavation of primary burials, by Alberto Rex González [4,5]. From 1979 to the most recent excavations in 2015, a total of 77 units (~314 m^2^) were opened in the AS2 site, including shovel tests and 3 long trenches (S1 Fig).

**Fig 2:**
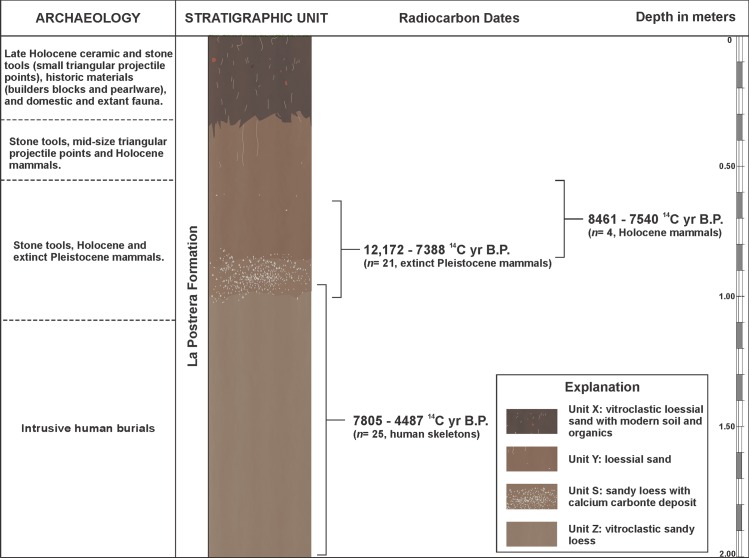
Generalized stratigraphic profile of the AS2 site.

The main objective of this paper is to present and evaluate the information regarding the earliest human occupation of the site (early to late Holocene occupations will not be discussed here). As a result of intensive biological, geological, and cultural processes, the AS2 site has a low stratigraphic resolution, as opposed to a high resolution which refers to strong preservation of artifacts and integrity of their spatial configurations [6]. The deposits of the sedimentary sequence are affected by dynamic process during or after their accumulation. These are subject to a variety of changes related to biotic and abiotic factors which affected original sedimentological characteristics and the contained archeological material. In spite of its low resolution, the AS2 site has significant archaeological characteristics for understanding aspects of the historical and evolutionary trajectories of hunter-gatherer societies. First, the site presents an ample temporal scale of human occupation from ca. 12,170 ^14^C yrs B.P. to the 19^th^ century. This extensive chronological dimension in a relatively short stratigraphic sequence (~2 m) of loessial sediments assigned to the La Postrera Formation [7] (Fig 2) has been one of the main causes of its low archaeological resolution. Second, there exists a high diversity of archaeological materials which provide a broad spectrum for detailed analysis (lithic, bone, ceramic, etc.) [4]. Third, the site presents an exceptionally varied and abundant number of human burials (50 individuals and counting), dated between 7805 ± 85 ^14^C yrs B.P. and 4487 ± 45 ^14^C yrs B.P. (n = 25 dates) [8].

With more than 30 yrs of excavation and interdisciplinary investigation at the AS2 site, it is not the lack of research or material remains, but the intrinsic characteristics of the site that make it a low resolution assemblage. In this sense, we present all the information in the most objective way, with both the certainties and uncertainties. The following paper will evaluate and discusses the general interpretations of the Pleistocene occupation at the AS2 site based on the AMS radiocarbon (^14^C) dates and the intensive re-analysis of the archeological material and site stratigraphy. Identifying the initial evidence of human occupation has been one of the central objectives of investigation at the site. This site has the potential to discuss two major subjects in American archeology: the early human expansion, and the interaction between hunter-gatherers and extinct Pleistocene fauna.

## Geology

The inter-sierra plains are developed in the southeast of the province of Buenos Aires, in the “Positive Bonaerense” [9] between the foothills of the Ventania and Tandilia Hill ranges (see Fig 1). Previously cited by Frenguelli [10] as a morphological sub-area called the "Pampa Inteserrana” (inter-sierra pampas) [11], this plain presents maximum heights of 200 masl, which gradually descends east towards the Atlantic Ocean. From a geomorphological point of view, the area where the archaeological site AS2 is located corresponds to the Acretional Loess Plateau Unit. The landscape is represented by an undulating relict plain, composed of continental Pliocene-Late Miocene Sub-cycle deposits which are capped by a thick calcareous duricrust [11]. During the late Pleistocene-Holocene Sub-cycle, loess deposits formed a continuous mantle across the Pampa Interserrana and the aeolian sands developed dunes in areas close to the sea [11]. While in low or depressed areas, fluvial-lacustrine deposits accumulated.

**Fig 3:**
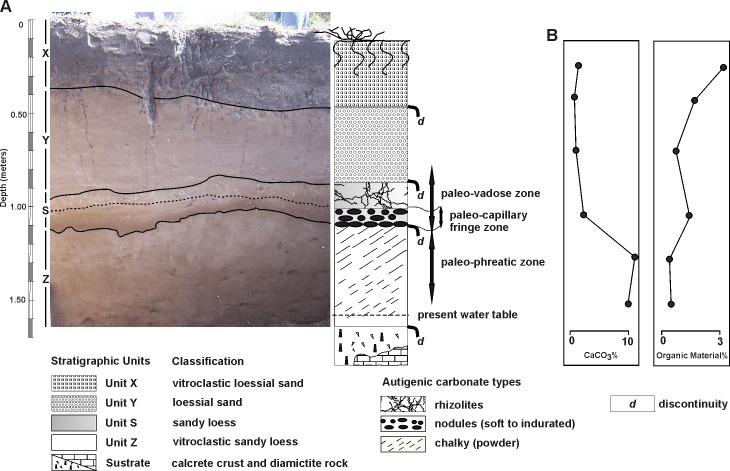
Geological context of the Arroyo Seco 2 site. (A) Stratigraphic profile from excavation Unit 70 (south wall). (B) Calcium carbonate (CaCO_3_) and organic material percentages.

The sedimentological analyses at the AS2 site have allowed the identification of four stratigraphic units, in concordance with the profile previously described by Fidalgo et al. [5] and Gentile [7], defined from the base to the top of the sedimentary profile as Z, S, Y, and X (Fig 3 and S2 File).

Unit Z: This unit lies unconformably on the calcrete crust developed in the diamictite (lithified sedimentary rock) consolidated sediments of the Late Miocene-Pliocene Sub-cycle [11]. Thickness of the Unit Z varies between 0.4 and 0.7 m. It has a pinkish gray loess color (5 YR7/2), composed of sandy silt. The sand content is below 50% of the total sample. The mean (Mz) value of 4.7 phi units is similar in both samples collected (bottom and top of the unit). The organic matter content is low; as such, this unit has the highest values of calcium carbonate for the entire stratigraphic sequence. The presence of abundant authigenic phosphate micro-nodules was recognized under the polarizing microscope. Powdery autigenic calcium carbonate is presented like a caliche (chalky zone). Towards the top of this unit, a layer between 0.12 and 0.17 m was identified with the highest concentration of carbonate, which makes coloration of this part of the column a bit clearer (see Fig 3). Strong cementation between grains does not exist, so the material has a loose consistency.

Unit S: This unit is recognized as a sandy-brown (7.5 YR5/2) loess deposit with varying thickness between 0.2 to 0.25 m. It is composed of sandy silt with very fine sand modal fractions and a mean coefficient (Mz) value of 4.55 phi units. The sand fraction percentage is less than 50% of the total sample. At the macroscopic scale, there are traits of incipient pedogenesis. This unit also presents features of postdepositational precipitation of calcium carbonate, represented by abundant soft to indurate nodules which form a nodular area (nodular zone) with abundant rhizoliths in the upper part of the unit. Its limit with the overlying Unit Y is sharp and wavy. Trench excavations around the site (see Fig 1 and S1 File) revealed that the development of the Unit S is restricted to the middle and upper sections of the knoll.

Unit Y: This unit is a homogeneous deposit of brown sandy loess (7.5 YR5/2) with variable thickness between 0.3 and 0.4 m. It consists of a silty sand deposit. The sand fraction percentage exceeds 50% of the total sample. No authigenic precipitates of calcium carbonate were found in the mass. The mean coefficient (Mz) value recorded was 4.12 phi units. Fresh volcanic glass shards are also present, but the increment of metamorphic lithic clasts (quartzite) minimizes the percentage. This unit passes in transition to the higher Unit X through a gradual and irregular contact.

Unit X: This unit is represented by a dark brown (7.5 YR3/2) to dark grayish brown (2.5 YR4/2) loess, represented by a silty sand deposit. The mean coefficient (Mz) has a 3.98 phi value. This unit is affected by present-day pedogenic processes.

The compositional and mineralogical analysis of the four stratigraphic units allowed defining a volcano-pyroclastic mineralogical association characterized by abundant volcanic glass shards and plagioclases (twinning and zoned). Volcanic and metamorphic lithic clasts are also present, with quartz as a minor component. Among the most abundant heavy mineral suites, 1–2% has been recognized as opaques, amphiboles, orthopyroxenes, clinopyroxenes and micas.

The features defined here correspond to those of other loess units studied in the inter-sierra plains [12,13]. The major differences found between the units of the AS2 stratigraphy is in the relative frequency of volcanic glass (dense shards and pumice) clasts, from freshly to slightly altered states. The relative abundance of this component is higher in the Units Z and X.

The stratigraphic sequence is composed of late Pleistocene Holocene fine aeolian deposits, represented by a loess mantle. It covers an undulating paleorelief composed of consolidated sediments (grouped under the collective name “Pampean Sediments”) of the Late Miocene-Pliocene Sub-cycle [11]. The sedimentary sequence is made up of four loess units, each one representing aeolian episodes separated by discontinuities. In three cases, erosive characteristics were found. In the case of the contact between the Units Z and S, it could only be defined as a stratigraphic unconformity, given the effects of meteoric diagenesis which has engaged these two units, masking the previous depositional features.

The Units Y and X present a higher sand fraction content (> 50%) and are defined as loessial sands; while the Units S and Z are referred to as sandy loess, following the proposal of Zárate and Blasi [12,13]. The stratigraphic units were classified, based on the particle size of these deposits and the mineralogical association, mainly by the abundance of volcanic glass. Thus, Units Z and X can be classified as a vitroclastic sandy loess, and a vitroclastic loessial sand respectfully. Unit Y can be classified as a loessial sand, and Unit S as a sandy loess unit.

Additionally, postdepositational reorganization by pedogenesis, and calcium carbonate precipitation by meteoric diagenesis, were observed affecting at least two of the stratigraphic units (Z and S). The first pedogenetic process is the result of moments of stability in the landscape, under wetter climate conditions, which favors the development of vegetation cover and a pedogenetical reorganization of the sediment. The second diagenetic process is more related to fluctuations in the paleo-water table, the groundwater chemical composition, the water depth from the surface, and the generated meteoric diagenesis produced at the expense of evaporation "per ascensum" (not pedogenic carbonates) [14–17]. The formation of certain morphologies of carbonates associated with pedogenic processes is not excluded. The precipitation of calcium carbonate in specific sectors of the stratigraphic sequence and under its different states (chalky, nodular, rhizolith zones) is reflected in the calcarerous profile. The carbonate related to the phreatic zone (chalky) and the vadose zone (nodular, rhizolith zones) can be differentiated in this profile.

The first and second aeolian episodes (Z and S) likely occurred during the arid phases of the Late Pleistocene coinciding with the Last Glacial Maximum period. The last depositional event (Units S) took place after erosive episodes of unknown intensity and duration. The deposit of Unit S is possibly related to the deflated material of the Unit Z mixed with other local dust-storm sediments. Later, the existence of a stability interval is presumed based on the incipient pedogenetic reorganization of Unit S, which would have taken place during the Late Pleistocene-Early Holocene under more humid conditions [18]. The third aeolian episode occurred at the Early Holocene, with the accumulation of Unit Y; which according to its mineralogy could be related to accumulations of local sands by deflation of nearby source areas. A marked reduction in the aeolian activity is shown at the regional scale for this time period [18].

Furthermore, a rise in regional groundwater level would have also occurred, related to the marine ingression during the Holocene which peaked at 6000 yrs B.P. [19]. Around 5000–4500 yrs B.P., the influence of an arid climate [20] led to the precipitation of a chalky authigenic calcium carbonate in the pheatic zone, and nodular and rizoliths in the vadose zone [14]. The meteoric diagenesis, jointly affected the Units Z and S.

The present shallow depth semiconfined aquifer in this area flows at 1.7 to 2 m under the ground surface. It is confined by low-permeability layers conformed of calcrete duricrust and indurated Pampean Sediments bellow the loess mantel. This substrate acts as an aquitard and as a regional source of alkaline surface and groundwater. According to the chemical characteristic of the present aquifer, it may produce dissolving and re-precipitation of the previous authigenic carbonates through time. It is possible that the calcium carbonate precipitated during the Middle Holocene in the Units Z and S are partially dissolved or re-precipitated as an effect of the current fluctuations in the phreatic. Finally, the Unit X is related to aeolian deposits connected to Late Holocene arid climate conditions, and the reworking by current agricultural activity.

## Material Analysis

### Spatial Distribution

Vertically, archaeological material starts just below the ground surface and extends to approximately 1.85 m. The Unit X includes lithic artifacts (small triangular stemless points), extant fauna and pre-Hispanic pottery, indicating a Late Holocene hunter-gatherer occupation. This unit also contains mid-19th to early 20^th^ century artifacts, such as pearl ware, glass, builder’s stone, and livestock [21]. There are currently no radiocarbon dates from bone or other material in the Unit X

**Fig 4:**
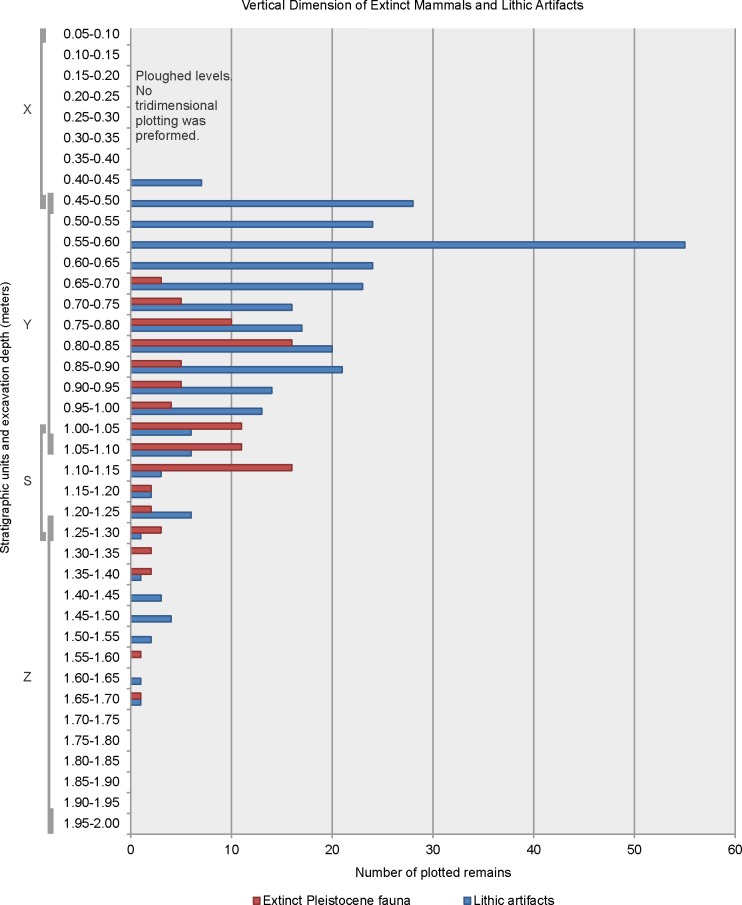
Vertical distribution of extinct Pleistocene fauna and lithic artifacts from the AS2 site. Calculated using the total number of tridimensional plotted remains from the excavation units 21–71.

The Unit Y contains the highest frequency of lithic artifacts and extant fauna remains which include guanaco (*Lama guanicoe*), pampas deer (*Ozotoceros bezoarticus*), nañdú (*Rhea americana*), small rodents, and armadillos. Extinct Pleistocene mammals begin to appear towards the middle and base of this unit (Fig 4). The lower section of Unit Y (staring from 0.75 m below the surface) contains the highest association of extinct Pleistocene fauna and lithic artifacts. Fourteen extinct Pleistocene mammal specimens were dated from the Unit Y, from 12,170 ± 45 ^14^C yrs B.P. to 9775 ± 45 ^14^C yrs B.P. The vertical dimension of those dated bone specimens are between 0.72 and 1.15 m below the surface. However, it is important to note that the contact between Y and S is undulating (see Fig 3) and therefore there are significant differences (more than 0.2 m) in the depth of this contact. This means that two specimens could be found at the same depth but belong to different stratigraphic units.

**Fig 5:**
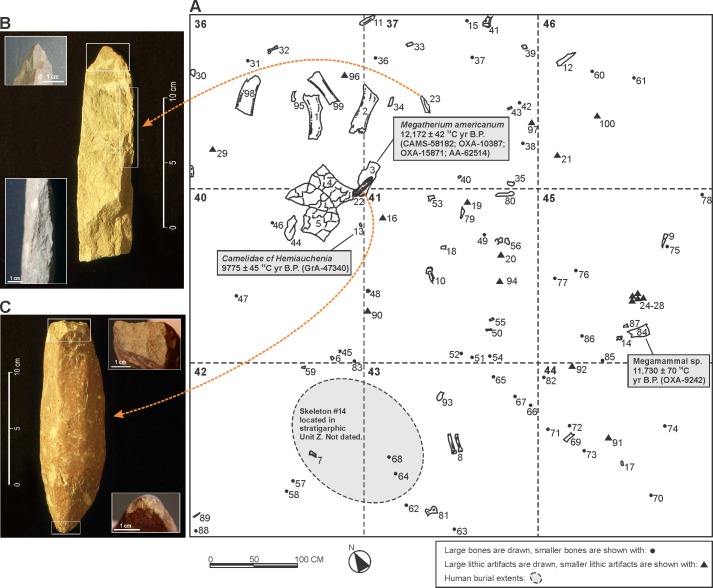
Example of the spatial association between bone, lithic artifacts, and human burials at the AS2 site. (A) Horizontal distribution map of central excavation units 36, 37, 40 to 46 (see S1 Table for map references). (B) Lutita artifact with detail of flaked edges. (C) Oval shaped cobble (rhyolite) with detail of fracture and rounded edge. Note: All material was recovered from the lower part of stratigraphic Unit Y with the exception of Skeleton # 14 (a single individual adult primary skeleton located in the lower stratigraphic Unit Z).

In the central excavation area, at the base of stratigraphic Unit Y (0.80 to 1.10 m below the surface), large sized flaked lithic artifacts, including a lutita (260 g) and a rhyolite (625 g) tool, are associated with a concentration of lower limb bones of *Megatherium*, *Hippidion*, *Equus*, and Camelidae cf. *Hemiauchenia* (Fig 5). The large lutita and rhyolite artifacts present rounded edges with fractures, modified by humans and likely used for disarticulating bones by high-energy impacts. Other lithic artifacts recovered in this context include a variety of tools made on non-local raw material such as quartzite, basalt rounded cobbles and ftanita [22]. While some of the lithic and extinct fauna materials would represent secondary associations, these large artifacts found only in this sector of the site and adjacent to megamammal lower limb bones are most likely a primary association. Given the lack of human burial activity and recognizable natural disturbance in this sector of the site, combined with the weight and size of the large stone tools and the limb bones of the extinct Pleistocene fauna, these artifacts are unlikely to have migrated along the stratigraphic profile.

The Unit S contains extinct faunal remains and the number of lithic artifacts decreases significantly. Three extinct Pleistocene mammal specimens were dated in the Unit S, from 11,250 ± 105 ^14^C yrs B.P. and 11,000 ± 100 ^14^C yrs B.P. (two *Equus*) to 7388 ± 74 ^14^C yrs B.P. (one *Eutatus*) (0.75 to 1 m below the surface). The human skeletons, which are intrusive, appear in the lower section of this unit (currently seven human burials), but only one has been dated in 6823 ± 69 ^14^C yrs B.P.

The Unit Z fauna remains and lithic artifacts are scarce. Bone fragments consist mainly of rodent. No extinct Pleistocene specimens have been dated in this unit; however, two guanaco specimens were dated, one in 8390 ± 410 ^14^C yrs B.P., and the other in 5793 ± 64 ^14^C yrs B.P. Both specimens are interpreted as bones which have migrated from the upper stratigraphic levels (postdepositational reorganization). This migration could be the result of human burial activity, as both specimens were recovered in excavation units with human remains; or natural processes, such as a rodent burrows. Twenty-six human burials, represented by both primary and secondary single-individual and multi-individual adults and infants from the Unit Z are dated between 7805 ± 85 ^14^C yrs B.P. and 4487 ± 45 ^14^C yrs B.P. Within this range of more than 3000 ^14^C yrs B.P., three burial episodes took place: Group 1, dated from 7805 ± 85 ^14^C yrs B.P. to 7580 ± 50 ^14^C yrs B.P. (13 individuals, with a vertical range of 1.5 to 1.9 m below the surface); Group 2, dated from 7043 ± 82 ^14^C yrs B.P. to 6300 ± 70 yrs B.P. (22 individuals, with a vertical range of 0.98 to 1.33 m below the surface); and Group 3, dated from 4793 ± 69 ^14^C yrs B.P. to 4487 ± 45 ^14^C yrs B.P. (2 individuals, with a vertical range of 1.11 to 1.46 m below the surface). Burial groups 1 and 2 were recovered from the main excavation area; while burial group 3 was recovered from peripheral excavation units (units 48, 52, and 59; see S1 Fig), where the thickness of the stratigraphic units were different. All burials recovered in the Units Z and S are considered intrusive from the overlying Unit Y, probably from the middle to upper sections. The lack of grave marks makes it difficult to clearly delineate the vertical and horizontal dimensions of the burials. No clear burial limits were recognized during the excavation.

Finally, it is important to mention that no projectile points were recovered in association with Pleistocene fauna. While some of the bones and artifacts are likely compromised by post depositacional human burial activity, and we do not interpret the example in Fig 5 as representing a discrete surface; the material remains from these excavation units suggests a *locus* of megamammal processing, where large sized artifacts were used to dismember skeletal units (*field-buthering units*) which were transported to the AS2 site and eventually fractured.

### Chronology

There are currently a total of 55 radiocarbon dates for the AS2 site. Twenty-five dates correspond to human skeletons (ca. 7805 to 4487 ^14^C yrs B.P.), twenty-one from extinct Pleistocene mammals (ca. 12,240 to 7388 ^14^C yrs B.P.), five from Holocene mammals (ca. 8461 to 5793 ^14^C yrs B.P.), three pedogenic carbonates (ca. 5740 to 1890 ^14^C yrs B.P.), and one from a Late Holocene paleosoil along the banks of the Tres Arroyos creek (1140 ± 60 ^14^C yrs B.P.). The majority of the material has been dated using the AMS technique. As many as eight different laboratories around the word have participated in the radiocarbon dating.

The 21 radiocarbon dates from extinct Pleistocene mammals (Table 1) are from 14 bone specimens that include: 6 genera (*Megatherium*, *Glossotherium*, *Toxodon*, *Hippidion*, *Equus*, and *Eutatus*); 2 families (Equidae, Camelidae cf. *Hemiauchenia*); and 2 indeterminate megamammal bones. Most of the extinct fauna are dated to the end of the Pleistocene; however, one specimen from extinct giant armadillo, *Eutatus seguini*, suggests survival into the Holocene (7388 ± 74 ^14^C yrs B.P., AA-90117). A comparable date of 7729 ± 48 ^14^C yrs B.P. (AA-85157, δ^13^C -20.3) [23] was obtained from organic matter of the sediment containing *Eutatus* bone remains at the Paso Otero 4 site (~130 km east of the AS2 site). The relatively small body size (ca. 200 kg), eating habits, and flexibility to survive in unstable environments of the Pampas are suggested to have favored this species survival into the Holocene [24].

**Table 1:** List of extinct mammal radiocarbon dates at the AS2 site.

Specimen	14C age BP	±	δ 13C value	F(δ 13C)	±F(δ 13C)	N%	Chemical fraction dated	Lab. Code	Bone Element	Fracture State	Storage Inventory Number	Excavation Unit	Stratigraphic Unit	Depth Below Surface	North coordinate	West coordinate	Current Resolution
*Equus neogeus*	12,170	45	-20.9	0.2198	0.0011	4.9	collagen	UCIAMS-142842	radius	fresh	FCS.AS2.14260	52	Base-Y	1.15 m	1.40 m	0.62 m	accepted
*Equus neogeus*	11,250	105	-22.1				collagen	AA-7965	carpel bone	dry	AS2.53.1	53	S	0.75 m	1.90 m	1.63 m	accepted
*Equus neogeus*	11,000	100	-20.1				collagen	OxA-4590	tarsal bone	dry	AS2.54.IX.12	54	S	0.77 m	0.84 m	0.51 m	accepted
*Equus neogeus*	8890	90	-25.0**				collagen	TO-1504	phalanx	dry	AS2.45.17	45	Base-Y/S	0.72 m	1.70 m	1.00 m	dismissed
*Eutatus seguini*	7388	74	-19.7	0.3986	0.0037		collagen	AA-90117	lumbar vertebra	dry	FCS.AS2.15265	69	S/Z	1.00 m	0.66 m	1.85 m	accepted
*Glossotherium robustum*	12,240	110	-20.6				collagen	OxA-4591	femur	dry	FCS.AS2.10239	66	Base-Y	0.85–0.95 m	1.50 m	1.55 m	uncertainty
*Glossotherium robustum*	10,500	90	-20.39	0.2705	0.0030		collagen	AA-9049	femur	dry	FCS.AS2.10239	66	Base-Y	0.85–0.95 m	1.50 m	1.55 m	uncertainty
*Megatherium americanum*	12,200	170	not informed	0.219	0.0044		XAD	CAMS-58182	tibia	fresh	FCS.AS2.8487	36-37-40-41	Base-Y	1.10 m	1.98 m	1.95 m	accepted
*Megatherium americanum*	12,170	55	-19.5			5.2	collagen	OxA-15871	tibia	fresh	FCS.AS2.8487	36-37-40-41	Base-Y	1.10 m	1.98 m	1.95 m	accepted
*Megatherium americanum*	12,155	70	-19.7			5.2	collagen	OxA-10387	tibia	fresh	FCS.AS2.8487	36-37-40-41	Base-Y	1.10 m	1.98 m	1.95 m	accepted
*Megatherium americanum*	11,770	120	-23.53	0.2309	0.0033		collagen	AA-62514	tibia	fresh	FCS.AS2.8487	36-37-40-41	Base-Y	1.10 m	1.98 m	1.95 m	accepted
*Megatherium americanum*	7320	50	-25.0**				collagen	TO-1506	tibia	fresh	FCS.AS2.8487	36-37-40-41	Base-Y	1.10 m	1.98 m	1.95 m	dismissed
*Megatherium americanum*	8470	240	uncorrected				collagen	LP-53	femur	dry	AS2.35/36.1	36–40	Base-Y	0.81 m	1.73–1.90 m	1.80–2.25 m	dismissed
*Megatherium americanum*	1800	110	uncorrected				collagen	SI-5481*	femur	dry	AS2.35/36.1	36–40	Base-Y	0.81 m	1.73–1.90 m	1.80–2.25 m	dismissed
*Camelidae* cf. *Hemiauchenia*	9775	45	not informed			12.6	collagen	GrA-47340	tooth (M2 right)	complete	FCS.AS2.10351	40	Base-Y	1.02 m	0.40 m	1.90 m	accepted
*Toxodon platensis*	11,750	70	not informed		0.0017		KOH	CAMS-16389	carpel bone	complete	AS2.54.VIII.20	53	Base-Y	0.74 m	0.27 m	1.30 m	accepted
*Toxodon platensis*	11,590	90	-16.5				collagen	AA-7964	carpel bone	complete	AS2.54.VIII.20	53	Base-Y	0.74 m	0.27 m	1.30m	accepted
Hippidion sp.	11,320	110	-20.8				collagen	AA-39365	3rd metatarsal	dry	FCS.AS2.9177	55	Base-Y	0.78 m	0.22 m	1.73 m	accepted
Equidae	11,190	110	-20.6	0.2483	0.0034		collagen	AA-90118	astragalus	complete	FCS.AS2.15000	70	Y/S	1.05 m	0.42 m	0.00 m	accepted
Megamammal sp. indet.	12,070	140	-15.3			5.7	collagen	OxA-9243	indeterminate	fresh	FCS.AS2.1987	55	Base-Y	0.79 m	1.30 m	1.52 m	accepted
Megamammal sp. indet.	11,730	70	-15.3			5.7	collagen	OxA-9242	indeterminate	fresh	FCS.AS2.10369	45	Base-Y/S	0.79 m	1.70 m	1.10 m	accepted

*Laboratories that are no longer operating or have changed their code designations.

**Standard correction (fixed).

Note: Bone specimens dated more than once are combined in the same row. The chemical fraction is stated as reported by the laboratory. Current resolution analysis of the radiocarbon chronology for the extinct Pleistocene assemblage is based on Steele and Politis [25].

An important point to emphasize is the discrepancies between dates from the same sample in different laboratories. In some cases, the variation can be as much as 5000 ^14^C yrs B.P. This variation is related to laboratory pretreatment of bone; specifically the methods used to extract bone collagen and remove secondary contaminants. The analysis of the radiocarbon chronology for the extinct Pleistocene assemblage performed by Steele and Politis [25] resolved many of the dating issues, and we can now effectively accept most of the dates (15 dates) and dismiss some (4 dates). The four dismissed dates include one specimen of *Equus neogeus* dated 8890 ± 90 ^14^C yrs B.P. (TO-1504), inconsistent with the determination on three separate equid samples; one specimen of *Megatherium americanum* dated 7320 ± 50 ^14^C yrs B.P. (TO-1506), discrepant with new high resolution AMS dates conducted on the same sample with four dates between ca. 12,200 and 11,700 ^14^C yrs B.P. One specimen of *Megatherium americanum* dated in 8470 ± 240 ^14^C yrs B.P. (LP-53) using standard methodology in 1980; with new dates from this taxon which suggests that this result is an outlier. The date in 1800 ± 110 ^14^C yrs B.P. (SI-5481) was dismissed because the laboratory reported an anomalous state of the extracted collagen; moreover, this date is extremely young and inconsistent with the accepted period for megafauna extinction in South America. There is still uncertainty with the sample of the *Glossotherium robustum* specimen (2 dates), which has dates 1740 ^14^C yrs B.P. apart (OXA-4591 and AA-9049). Currently, it cannot be determined which of these dates is the correct age of the *Glossotherium*.

### Lithic Material

**Fig 6:**
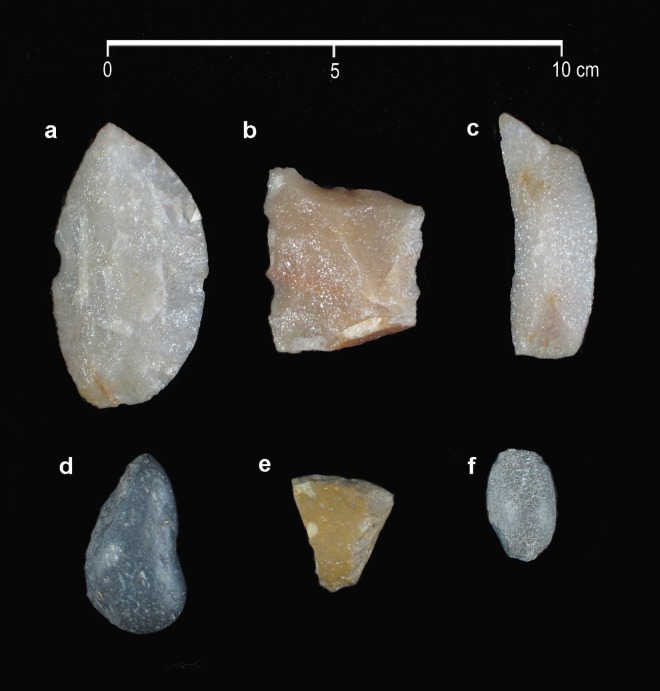
Sample of the lithic artifacts found in the levels associated with extinct fauna. (a) side scraper, quartzite; (b) retouched flake, quartzite; (c) retouched flake, quartzite; (d-e) scrapers made on coastal rounded cobbles; (f) bipolar cobble. All material recovered from the lower part of Unit Y, Unit S, and the upper part of Unit Z. Photograph modified from Leipus and Landini [27].

Analyzed lithic materials from the lower part of Unit Y, Unit S, and in the upper part of Unit Z (excavation units 21 to 66) show formal artifacts and thousands of flakes and lithic debitage (Fig 6 and S4 Fig) [26]. This sample is formed by 56 artifacts, cores and bipolar (80.3% quartzite) recovered from the lower part of Unit Y, and 47 artifacts, cores and bipolar (80.7% quartzite) recovered from Unit S and the upper part of Unit Z [27]. Formal tools include: two *piéce esquilleé* quartzite; a convergent quartzite side-scraper; two bipolar cores (one quartzite, and one basalt); a lateral-front quartzite scraper; a perimetral edge brush; and various lateral retouched quartzite artifacts [27]. Forty-three instruments from the lower part of Unit Y were microscopically analyzed, of which 29 (67.4%) represent traces of use. In Unit S, and the upper part of Unit Z, 32 artifacts were analyzed and 22 (69%) show traces of use [22]. In a sample coming from the excavation units 54 to 67 (which represent 26% of the total excavated area), 2030 lithic debitage were recovered in the lower part of Unit Y and 299 in the Unit S and upper part of Unit Z. Among these, 95% are quartzite, mostly from the Sierras Bayas Group [22]. The functional analysis performed by Leipus [22] demonstrates that a vast majority of the retouched pieces from the stratigraphic Units Y, S and Z were used for working wood and skin, while the working of bone and soft animal tissue are underrepresented. No plant material has been recovered at the site, such as wood, but evidence obtained from the functional analysis shows that wood was an important resource. With regards to the working of skins, a large majority of the flaked edges were used transversely on dry skin. Consequently, it is likely that the skins were brought to the site in a state of intermediate processing [22]. As mentioned above, no projectile points were found associated with the early components; however, eleven points were found with human burials dated in ca. 7800 to 7600 ^14^C yrs B.P., and another seven points were found in the upper levels of the site (S3 File).

All raw materials from the lithic artifacts are allochthonous. The majority (ca. 90%) is quartzite, mostly from the Sierras Bayas Group (in the Tandilia Hill range, ~110 km east of the site) (see Fig 1). Other raw materials include chert (*ftanita*), from the Tandilia Hill range (Sierras Bayas Group, Cerro Largo and Loma Negra Formations, ~150 km northeast of the site) and rounded basalt cobbles from the Atlantic coast (~56 km toward the south of the site).

### Extinct Pleistocene Bone

More than 100,000 faunal remains have been recovered from the AS2 excavations (90% of which correspond to bone fragments < 2 cm); approximately 6200 have been determined at a taxonomic level, and 40 different taxa were identified [28]. The total number of extinct Pleistocene mammal remains identified is currently 272 (Table 2). Considering the entire fauna assemblage, this ranks third behind *Lama guanicoe* (n = 892) and rodents (n = 480). A large part of the Pleistocene mammal bone assemblage corresponds to indeterminate specimens (73%; n = 199); which includes indeterminate fragments (n = 100) and detached pilosa dermal ossicles (n = 99). A total of 12 taxonomic categories have been assigned. This includes five ranked at the level of species [*Equus neogeus*, *Eutatus seguini*, *Glossotherium robustum*, *Megatherium americanum*, *Toxodon platensis*], four ranked at the level of genus [*Glypotodon* sp., *Hippidion* sp., *Macrauchenia* sp., *Mylodon* sp.], two ranked at the level of family [Camelidae cf. *Hemiauchenia*, Equidae], and one ranked at the level of subfamily [Lestodontinae cf. *Lestondon*]. There are four groups of giant ground sloth [*Glossotherium robustum*, *Megatherium americanum*, *Mylodon* sp., Lestodontinae cf. *Lestodon*], two groups of extinct horse [*Equus neogeus*, *Hippidion* sp.], two South American ungulates [*Toxodon platensis*, *Macrauchenia* sp.], one camelid [*Hemiauchenia*], one glyptodont [*Glypotodon* sp.], and one giant armadillo [*Eutatus seguini*].

**Table 2:** Quantification of anatomical units in the extinct Pleistocene mammal assemblage.

Anatomical unit	*Equus neogeus*	*Eutatus seguini*	*Glossotherium robustum*	*Megatherium americanum*	*Toxodon platensis*	*Glypotodon* sp.	*Hippidion* sp.	*Macrauchenia* sp.	*Mylodon* sp.	Camelidae cf. *Hemiauchenia*	Lestodontinae cf. *Lestondon*	Equidae	Megamammal sp.	Total
rib						1						1		2
molar	1									1		1		3
lumbar vertebrae		1												1
acetabulum												1		1
iliac crest											1			1
joint bone												1		1
astragalus										1		1		2
calcaneus	2													2
scaphoid	1													1
scapula												1		1
phalange	2	1		2		1	2	1				1		10
femur	1		1	2		1						1		6
humerus	1	1										2		4
lunate									1					1
magnum										1				1
metacarpal	1													1
metatarsal	2						1							3
metapodial	3							1		1		2		7
carpal bone	1				1		1					1		4
tarsal bone	2													2
pisiform										2				2
radius-ulna	1									1		2		4
ulna												1		1
patella							1							1
tibia	1		1	4				1		1		1		9
unciform										1				1
scute						1								1
dermal bone													99	99
indeterminate													100	100
Total	19	3	2	8	1	4	5	3	1	9	1	17	199	272

There is an important variety in the Pleistocene mammal body size [29,30], such that the assemblage can be divided into three size categories: 1) large-sized megamammals (>1700 kg), including *Megatherium americanum* (ca. 6000 kg) and Lestodontinae cf. *Lestondon* (ca. 3000 kg); 2) medium-sized megamammals (between 1700 and 1000 kg), which include *Glossotherium robustum* (ca. 1700 kg), *Mylodon* sp. (ca. 1000 kg), *Toxodon platensis* (ca. 1600 kg), *Macrauchenia* sp. (ca. 1000 kg), and *Glypotodon* sp. (ca. 1500 kg); and 3) large mammals (between 500 and 200 kg), which include the *Equus neogeus* (ca. 300 kg), *Hippidion* sp. (ca. 500 kg), Camelidae cf. *Hemiauchenia* (ca. 300 kg), and *Eutatus seguini* (ca. 200 kg).

The indeterminate fragments (n = 100) were classified as spongy and compact, and likely correspond to long bone of large or medium sized megamammals (≥1000 kg). In reference to the detached pilosa dermal ossicles (n = 99), these belong to any one of the four ground sloths mentioned above.

**Fig 7:**
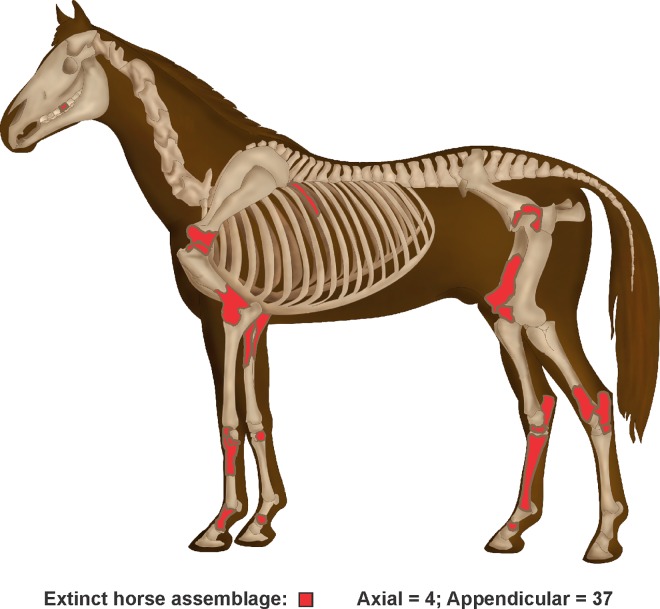
Skeletal representation of the extinct horse assemblage at AS2. Horse image modified from https://commons.wikimedia.org/wiki/File%3AHorseanatomy.png.

The most abundant taxon represented in the assemblage is *Equus neogeus* (n = 19; MNI = 1). Skeletal parts of this horse are mostly from the appendicular skeleton, including limb bones (tibia, humerus, femur, radius, metacarpal, and metatarsal), phalanges, carpal, and tarsal bone. Only one molar was identified from the axial skeleton. The other horse, *Hippidon* sp. (n = 5; MNI = 1), present fewer specimens; however, all elements belong to the appendicular skeleton, including metatarsal, astragalus, phalanges, rotula, calcaneus, and carpal bone. Taking into consideration the elements identified as Equidae (n = 17) (which may correspond to either of these taxa), the total number of appendicular and axial skeleton parts for extinct horse increases (n = 41), including the astragalus, scapula, ulna, rib, and teeth. With the exception of the molars and a single piece of rib bone and a piece of the acetabulum, the extinct horse assemblage is almost entirely made up of the appendicular skeleton (Fig 7). This is also the case with the remaining extinct Pleistocene bone assemblage, where a minimal number of fragments of skull, vertebrae and rib are present.

### Taphonomy

From the onset, it was clear that several processes were modifying the AS2 bone assemblage. During excavation, processes such as roots, rodent burrows, and human burials suggested perturbation at the site. When detailed taphonomic analysis was performed, a variety of bone modifications were identified (S2 Table).

**Fig 8:**
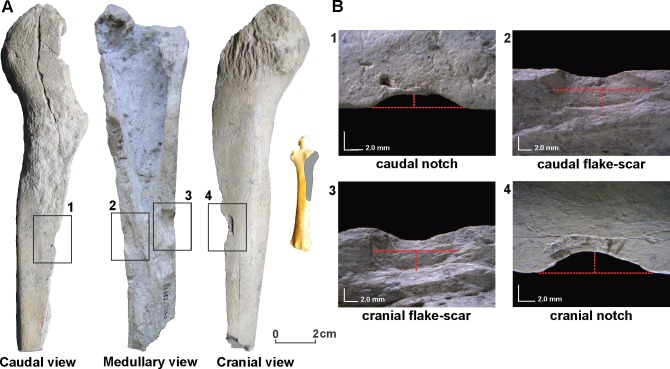
Radius of *Equus neogeus* dated 12,170 ± 45 ^14^C yrs B.P. (UCIAMS-142842). (A) Three views with location of impact. (B) A detailed view of notches and flake-scars; dotted lines illustrate the maximum depth and breadth of notches and flake-scars.

The majority of the extinct Pleistocene bones are fractured in a dry state (83%). At least 5% of the bones present evidence of fresh fractures; however, very few associated technological features resembling a hammerstone and anvil strike (e.g., apparent striking platforms, bulb of force) are observed on these (S2 Fig). The most probable explanation is that most of the fresh fractured bones were broken by humans, and as a result of several natural modifications, have lost some of their technological features. There is however other potential processes, such as carnivore activity, trampling, or post-deposit sediment loading which could be responsible for bone fractures [31–33]. The best example of anthropic fracturing at the site is a radius from *Equus neogeus* (dated 12,170 ± 45 yrs B.P.; UCIAMS-142842) that presents robust evidence of hammerstone bone breakage (Fig 8). The shape and color of the fracture surface, combined with the presence of associated technological features (two impact points with their respective notches on opposite sides of the shaft), indicates that this bone was intentionally broken while still fresh. When the notches are analyzed (Table 3), measurements show large and shallow shapes, most likely resulting from dynamic loading rather than static loading [34–38]. According to actualistic studies [34–36], the ratio measurements on the notch shapes and platform angles fall within the range of variation for notches produced by a hammerstone. They do however also fall within the range for notches produced by a large carnivore the size and morphology of a lion or hyena (two predators not found in the South American fossil record). With just one sample in the AS2 site, we are unable to statistically compare dimensional values; however, the lack of other carnivore related marks on this particular specimen (punctures, crenulated edges, furrowing, etc.), and the low percentage of carnivore marks in the remaining bone assemblage (1.2%; see taphonomic information below) strongly suggest an anthropogenic fracture.

**Table 3:** Measurements on notch shapes and platform angles from the radius of *Equus neogeus* dated 12,170 ± 45 ^14^C yrs B.P. (UCIAMS-142842).

	Caudal view		Cranial view	
	Flake-scar	Notch	Flake-scar	Notch
breadth (mm)	14.43	9.71	12.69	14.28
depth (mm)	2.52	1.38	3.88	2.49
radio	5.73	7.03	4.4	5.72
platform angle		107		85

A potential process which can cause dry bone breakage is weathering, a modification observed in 54% of the Pleistocene bone. Most of the specimens present an advanced stage of this modification [stages 3 to 4, according to Behrensmeyer [39]]; particularly in the large and medium megamammals like *Glossotherium robustum*, *Megatherium americanum*, *Toxodon plantensis*, and *Macrauchenia* sp. (S3 Fig). Smaller sized fauna like extinct horse and *Eutatus seguini* were less affected. This suggests that part of the extinct Pleistocene assemblage, particularly the larger sized taxon, were exposed to the environment for prolonged periods of time. It is also possible that some of the bones were re-exposed during Early and Middle Holocene human burial activity, or some natural geological process since unconformities were identified in the profile. The bone weathering contributed to the decay of the cortical surface, and in advanced stages led to bone fragmentation. In most cases, the weathering is continues along the fracture surfaces, indicating that the bones were fractured before they were weathered.

Calcium carbonate (CaCO_3_) also affected many of the bones (51%). Unlike weathering, the distribution of this modification does not appear to have favored any particular body size. All of the bones, with the exception of *Glyptodon* sp. presented chemical sedimentary deposits of CaCO_3_. The calcium carbonate has both a positive and negative outcome on the bone. On the one hand; when dense, it contributes to the preservation of specimens. On the other hand, it can mask features such as cut marks or fractures. The majority of the specimens however presented only a light coating.

A modification which had a destructive effect on the preservation of the bone surface is chemical deterioration (46%). This modification occurs as a result of multiple processes in the micro-environment where the bones are deposited. Possible causes are the concentration of acids in the soil, bacteria, or the growth and activity of roots. Here, all of the taxa, with the exception of *Eutatus seguini* and *Macrauchenia* sp. presented evidence of chemical deterioration. The bone alteration of this process decreases the possibilities of recognizing cut marks and contributes to the overall breakdown of the bone structure [40,41].

Directly related to chemical deterioration is root action, which is evident in a significant part of the sample (39.9%). Root etching is continuous on the surface of the bone. It occurs both on the fracture and non-fracture surface, suggesting that this process happened after the bone was fractured. Root action would not have caused any spatial displacement of the material; however, it does have destructive effects on the surface of the bone [42]. Intensive root etching masks other surface modifications. It can also lead to the collapse of the bone structure, and when combined with other modifications, eventually cause fragmentation.

A second type of chemical deposit registered in the bone assemblage is manganese staining (30.7%). In extreme cases, the entire cortical surface may be covered by manganese bonded to the bone surface, completely masking other modifications. While all but two taxa were affected by manganese staining, the intensity was mild, having minimal effect on the ability to identify other types of modifications. It was also continues on the bone surface, suggesting that this processes occurred after the bone was fractured.

Agents that can spatially modify the sample are rodents (35.5%). The rodent action does not appear to have favored any particular taxa. All specimens contain evidence of rodent gnawing except *Glossotherium robustum*. Several rodent skeletal remains were found in the site, including *Dolichotis patagonum*, *Lagostomus maximus*, which are large extant rodents between 8 and 5 kg [43,44], capable of significant bone damage. For example, a bone specimen of *Toxodon platensis* presented rodent damage likely from one of these species (S3 Fig).

Only two specimens (both from Equidae) were identified with carnivore marks (1.2%), and one of these specimens presented large puncture depressions (S3 Fig). All three punctures were measured using the methods described by Delaney-Rivera et al. [45], with an average measurement of 7.383 mm long (major axis) and 5.727 mm wide (minor axis). Once again, the sample size makes it difficult to compare statistically; however, the size of these marks exceeds those described by puma on extinct faunal samples from Patagonia [46]. With just two specimens, carnivores are not considered an agent that has participated significantly in the accumulation or fragmentation of the bone assemblage.

Five specimens were identified with evidence of geological abrasion (3.1%). Given the sedimentary context of the site, the likely cause is aeolic. Four of the five specimens were identified as indeterminate megamammal specimens. One specimen of *Equus neogeus* presented exposed cancellous bone tissue along the edge of a fractured surface which appears to have been caused by geological abrasion preceding the fracture.

Just one specimen of extinct Pleistocene fauna was registered with thermal alteration (0.5%). The specimen is an indeterminate megamammal bone.

In summary, the natural taphonomic modifications registered on the extinct Pleistocene fauna include a significant degree of weathering, chemical deposit (in particular CaCO_3_) and chemical deterioration. There are moderate levels of root etching and manganese staining, and minimal evidence of carnivore activity. The joint alteration of some of these processes likely contributed to the fragmentation of bones, while others favored bone preservation. The natural modifications observed on the fracture surface were continuous with the rest of the bone, suggesting that most of the taphonomic processes occurred after the bone was broken. These results imply that bone was likely transported by hunter-gatherers, and many of the specimens were fractured before they experienced post-deposit damage.

## Discussion

Considering the data from radiocarbon dates (see Table 1), Pleistocene fauna death occurred in the AS2 site between ca. 12,200 and 7300 ^14^C yrs B.P. Within this range, at least four different dates-of-death occurred: (1) *Megatherium* and *Equus*, ca. 12,170 ^14^C yrs B.P. (13,975–14,152 cal yrs B.P.); (2) *Toxodon*, ca. 11,750 ^14^C yrs B.P. (13,473–13,594 cal yrs B.P.); (3) *Equus* and *Hippidion*, ca. 11,182 ^14^C yrs B.P. (13,035–13,100 cal yrs BP); and (4) *Eutatus*, ca. 7388 ^14^C yrs B.P. (8175–8208 cal yrs B.P.). In order to discuss the human signal in the lower part of the stratigraphic Unit Y and S, where the narrowest association between Pleistocene fauna and lithic artifacts are observed, four lines of evidence need to be deliberated: 1) the spatial and stratigraphic association of archaeological material; 2) the selection of extinct fauna skeletal parts; 3) the concentration of Pleistocene species in a restricted area with positive landscape features; and 4) the human processing features in extinct fauna bones.

*1) Spatial and stratigraphic association of archaeological material*. Dated extinct Pleistocene bones associated with a variety of lithic artifacts and debris are scattered in the stratigraphic units Y and S, from 0.72 to 1.3 m below the surface. However; despite the close association of large sized flaked lithic artifacts, including a lutita and a rhyolite tool, with a concentration of lower limb bones of *Megatherium*, *Hippidion*, *Equus*, and *Camelidae* cf. *Hemiauchenia* (see above), no discrete horizons or paleo-surfaces have been detected (see Fig 4). The radiocarbon dates from extinct fauna in the lower part of the stratigraphic units Y and S demonstrate an important chronological dispersion; however the delimited horizontal and vertical spatial association in specific sectors of the site supports a close relation between Pleistocene fauna and lithic artifacts. What is important to make clear is that like any multi-component site with an ample chronological range and short stratigraphic sequence with low sedimentation rate, there is a high grade of complexity in its formation. While the action of variable and constant post-deposit processes has been studied in detail from the beginning of the systematic research at the site [40], understanding the formation processes of the archaeological assemblage in the site has been one of the central interests in the research projects and an important part of current study. Given the spatial and temporal resolution of the human burials, as well as the pre and post occupations related with faunal processing, the site represents a type of cumulative palimpsest (*sensu* Bailey [47]); however, one with discrete and rapid episodes of anthropogenic burials. In other words, the site represents successive episodes of occupations, superimposed one on top of the other without much loss of material, but with low resolution of spatial organization. The formation of the palimpsest is recurrent in the deposit of archaeological materials [48,49]; and in general, the palimpsest sites can offer very useful information regarding the formation processes [47], and eventually an estimation of the magnitude of the post-deposit disturbance can be made. With this in mind, the significance of the site, nor its importance in addressing some of the central topics of the historical trajectory and evolution of the Pampean indigenous populations, should not be underestimated [50].

*2) Selection of extinct fauna skeletal parts*. Bone quantification shows a higher representation of the appendicular skeletal parts over the axial skeleton. Very few axial skeletal parts from identified species correspond to fragments of skull, vertebra, acetabulum and rib. This assemblage is comparable with deposit and transport strategies of hunter-gatherers as shown in ethnographic studies. For example, the Efe and Lese groups hunt elephants cooperatively in the northeast of the Ituri Rainforest in Africa. While there is a variable pattern in terms of the amount of bone transported to the campsite, some bone elements are never processed (and thus never transported): skull, mandible, scapula and innominate bone [51]. Another interesting ethnographic case is the Hadza from the savannahs of Africa, which is environmentally closer to the Pampa grasslands during the end of the Pleistocene, in terms of the diversity of fauna and size of prey. This ethnographic group transports the entire carcass from the kill site to the residential campsite, with the exception of the larger sized animals (giraffe, and elephant) [52]. Both types of methods used to transport carcasses can generate sites in where one of the principal activities was the processing of carcasses; deposits comparable to the Pleistocene bone assemblage at AS2 site.

While it is possible that bone mineral density has influenced the representation of skeletal parts [53], it does not appear to be an important factor in the differential bone survival. There is a remarkable under-representation or even absence of certain bone elements with high density values such as teeth and cranial parts like tympanic annulus and occipital condyle. Most of the larger size megamammals, such as the *Megatherium* would have been hunted or scavenged close to the site, and its parts transported later. Given the body mass of this species (between 4 and 5 tons) [30], it would have been extremely difficult to transport the entire carcass and even challenging to transport complete hindquarters weighing between 600 and 750 kg, and forequarters weighing between 250 and 300 kg. Taking into consideration these values, the best hypothesis is that the *Megatherium* was hunted or scavenged near the site, the skeleton was butchered into smaller parts, and these units were then transported to their current location at the site. The larger bones were transported with portions of meat already removed, and the bone may have been used for other purposes such as bone quarrying [54,55]. The under-representation of some anatomical skeletal elements supports the human action, through the selection of determined parts of the carcass based on the size of the prey, as one of the principal agents in the formation of the Pleistocene fauna deposit. The anatomical representation of megamammals appears to be the result of differential transport of items based on yield of meat or bone. In this sense, the low frequency of dental material appears to be the consequence of discarding of the skulls at the hunting/scavenging site.

*3) Concentration of Pleistocene species in restricted areas*, *with positive landscape features*. In the ca. 100 m^2^ of the main excavation area, at the upper sector of the knoll, 11 taxa classifications have been assigned to Pleistocene fauna. During the temporal lapse between ca. 12,170 ^14^C yrs B.P. and 11,180 ^14^C yrs B.P. there is evidence of *Megatherium*, *Toxodon*, two extinct horse, and probably *Glossotherium* bone deposit. The probabilities that the Pleistocene fauna deposit are not of anthropic origin and that the site functioned as a place of natural death, a “bone trap” of Pleistocene species, are very low. Besides the fact that the bone assemblage appears to be the result of differential transport, the deposit is located on a positive landscape feature which is currently ~2.6 m above the level of the lake floor (see Fig 1). The reconstruction of the paleotopography indicates that this difference would have been even greater during the Late Pleistocene, a period when the shallow lake was active: between 4 and 5 m, and that a greater degree in the slope of the knoll would have existed [7]. The characteristic of the landscape does not favor the hypothesis that the knoll functioned as a type of “bone trap” [56]; which, in general, are found in depressions of the landscape like rivers and streams [57] or other bodies of water [58]. Furthermore, the limited vertical dimension of the extinct Pleistocene bone in the AS2 site assemblage suggests that the accumulation is discontinuous. In other words, if megamammal bones were being deposited naturally at the site, one would expect to find more bone specimens in the lower stratigraphic Unit Z, which formed during the Late Pleistocene. According to the regional geological record, the formation of the stratigraphic Unit Z may have started ca. 19,000 to 20,000 years BP [7] during which time the maximum diversity of mammals is recorded in Pampas [59]. Another aspect to consider is how, at a regional scale, these types of loess knolls do not behave as natural accumulators of bone. While there are currently no systematic regional paleontological studies which discuss this, local observations suggest that the bone concentration at the AS2 site is very high, and the variety of species represented is greater than any natural accumulations of bones usually found in the aeolian *La Postrera* Formation deposits in the Pampean region [60]. Therefore, the density and diversity of the Pleistocene bone remains in AS2, favors the action of hunter-gatherers as the principal accumulators of the deposit.

*4) Human processing features in extinct fauna bones*. The AS2 bone assemblage is highly fractured, the majority of which are classified as dry fractures. The frequency of these dry fractures is a consequence of various taphonomic processes which over long periods of time breaks down bone and cause fragmentation [40]. The identification of specimens with fresh fractures implies dynamic (human-hammerstone) force on the bone; although static (carnivore-bite) force cannot be discarded. However, while large carnivores with bit force similar to hyena and lions existed towards the end of the Pleistocene in southern South America [61], there is minimal evidence to suggest their interaction in the AS2 site. In reference to medium sized carnivores such as the puma (*Puma concolor*), further experiments are needed to confirm if this predator was in fact capable of producing similar sized notches. Therefore, while some ambiguity in fractured bone will always exist, and normally will require a more direct type of evidence (i.e, cut marks) to ratify the human origin, the information presented here helps to reduce much of this ambiguity and sustain the anthropic fracturing of the fresh fractured specimens. Moreover, one specimen of *Equus neogeus* bone (a proximal right radius fragment) dated at 12,170 ± 45 ^14^C yrs B.P. presents strong evidence of anthropic fracturing with associated technological traits (see Fig 8). Finally, the absence of cut marks is likely related to the extensive natural modifications (weathering and chemical deposits) which mask or destroy this type of evidence [40]. On the other hand, the lack of marks may be the consequence of a butchering strategy of megamammals, since the amount of soft tissue would require little contact with the bones cortical surface. As demonstrated in actualistic studies, large quantities of meat from large sized prey can be filleted without making contact with the bone [62,63]. Use-wear analysis in the associated lithic artifacts in the AS2 assemblage is consistent with this hypothesis; as only three artifacts present evidence of bone contact [22].

## Conclusions

While each one of the four lines of evidence resumed and discussed previously could be explained in some way without a human intervention (see for example the discussion in Haynes [64]); the combination of the four lines of evidence suggests that the hypothesis more parsimony (*Occam's razor*) is that which situates the human action as a central causal factor, however not exclusive, in the formation of the extinct fauna bone assemblage and in its association with lithic artifacts. The information discussed here supports the following model of cultural formation for the early deposits at the AS2 site. The earliest human signal is situated in ca. 12,170 ^14^C yrs B.P. (14,064 cal yrs B.P.) In this time period, occupants hunted/scavenged an extinct horse (*Equus neogeus*) and giant ground sloth (*Megatherium americanum*), probably along the border of the temporary lake (or another body of water) located near the site. The butchering began with the processing of the hind limbs in this plausible nearby location; which, when separated in smaller anatomical units (*field-butchered units*) were transported to the top part of the knoll where the processing was later finished. This area would have functioned as a *short-term campsite/carcass processing site* (*sensus* Fisher Jr.[51]). There, some anatomical units were disarticulated and the larger bones were broken using large stone tools. Some smaller artifacts were also used to cut and process the skins. The hunting/scavenging events of the early hunter-gatherers at the AS2 site were likely repeated several times. Temporal campsites were installed in the area for the butchering of *Equus* and *Hippidon* at ca. 11,180 ^14^C yrs B.P. Given that these are smaller bodied animals (< 500 kg), the carcasses entered into the site more complete, and the anatomical units included elements like rib, teeth, and a piece of pelvis. During this period, other species of megafauna (*Toxodon*, *Hemiauchenia* and *Glossotherium*) were transported to the site, although the evidence of human agency is still vague for these taxa. Between these events, the AS2 site was likely visited by carnivores which scavenged some of the bone remains, though evidence for this activity is minimal. In conclusion, while there is a high degree of complexity in the formation of the AS2 site, the first evidence of human occupations dated between ca. 12,170 ^14^C yrs B.P. and 11,118 ^14^C yrs B.P. (14,064 and 13,068 cal yrs B.P.) are interpreted as a succession of transitory extinct mammal processing campsites; which formed a type of palimpsest and consequently cannot be differentiated among each other.

The hunting of Pleistocene fauna in America is a heavily debated topic [65–68]. Generally speaking, in North America there is a wide agreement concerning the human predation of mammoth (*Mammuthus* spp.), mastodon (*Mamut americanum*) and bison (*Bison* spp.). Less conclusive is the exploitation of American horse (*Equus* sp.) [69], extinct camelid (*Camelops* sp.) [70] and the giant ground sloth (*Magalonix jeffersonii*) [71]. For South America, the exploitation of Pleistocene fauna has been proposed for mastodon [72], American horse (*Hippidion saldiasi*, *Equus*) [72–74], giant ground sloth *Megatherium americanum* [75], *Doedicurus clavicaudatus* [76] and possibly *Hemiauchenia* sp. [77] and *Eutatus seguini* [23]. With respect to *Megatherium*, AS2 is added to a list of sites which include the Campo Laborde site, in Argentina [75], and tentatively also to the El Vano site, in Venezuela [78] where *Eremotherium rusconii* remains were found. In the case of the two species of extinct horse, the evidence of AS2 adds to the wealth of important data that supports the human consumption of horse in various South American sites [72,73].

Finally, like other early sites, the first signal of human occupation in AS2, dated in ca. 12,170 ^14^C yrs B.P. supports that Clovis was not the first human population in America (for a recent review see Madsen [2]). The second human signal in AS2, dated in 11,200 ^14^C yrs B.P. is contemporary with the initial occupations of Clovis [1,79]. However, nor AS2, or other sites from Argentina and the rest of the Southern Cone [80–82] have ages various millennium older than Clovis, and despite recent claims [83], there is not yet a robust human signal firmly dated older than 13,000 ^14^C yrs B.P. The evidence presented here supports a hypothesis of *Homo sapiens* dispersion in the Southern Cone before Clovis but after the Last Glacial Maximum (LGM). The AS2 site, along with other sites from Patagonia (such as Piedra Museo, Cueva Casa del Minero, and Cueva Cerro Tres Tetas, see discussion in Steele and Politis [25]), supports the idea of a human occupation previous to the transmission of the fish-tail projectile point technology; which in the Southern Cone is dated between 11,000 ^14^C yrs B.P. and 10,000 ^14^C yrs B.P. [82,84,85]. The absence of these projectile points in the AS2 site is in agreement with the chronology for this type of technology in the Southern Cone. The age of the AS2 site is consistent with this model, and that of a first pulse of entering of the peopling in America between 17–15 ka cal B.P. and posterior to the onset of the deglaciation of the Last Glacial Maximum (LGM) [86]. In this sense, the arrival of *Homo sapiens* into the Southern Cone at 14,000 years ago represents the last step in the expansion of modern humans throughout the world and the final continental colonization.

## Supporting Information

S1 Fig Excavation grids at the Arroyo Seco 2 site (2015). (TIF)

S2 Fig Examples of extinct Pleistocene bone with evidence of fresh fractures. (A-C) indeterminate megamammal fragments (not dated).
(TIF)

S3 Fig Examples of extinct Pleistocene bone with extensive natural modifications. (A) indeterminate megamammal fragment with extensive weathering (not dated). (B) ulna from Equidae sp. with close-up of carnivore punctures (no dated). (C) carpal bone of *Toxodon plantensis* with weathering and large rodent marks, dated 11,750 ± 70 yr B.P. (CAMS-16389) and 11,590 ± 90 yr B.P. (AA-7964).
(TIF)

S4 Fig Quartzite artifacts from the base of Unit Y and Unit S. (A) retouched flake (Unit S; 1.08 m below surface; FCS.AS2.11247); (B) retouched flake (Unit Y; 0.76 m below surface; FCS.AS2.11247); (C) retouched flake (Unit Y; 0.77 m below surface; FCS.AS2.11351); (D) retouched flake (Unit S; 1.03 m below surface; FCS.AS2.11257); (E) core (Unit Y; 0.70 m below surface; FCS.AS2.11280); (F) core (Unit Y; 0.90 m below surface; FCS.AS2.11250); (G) core (Unit S; 1.05–1.10 m below surface; FCS.AS2.11272); (H) unknapped flake (Unit Y; 0.90 m below surface; FCS.AS2.11354); (I) unknapped flake (Unit Y; 0.70 m below surface; FCS.AS2.11369); (J) unknapped flake (Unit Y; 0.70–0.75 m below surface; FCS.AS2.10218). Note: dotted lines indicate artifacts edges, and arrows point to the worked edges of the retouched flakes.
(TIF)

S1 File Google Earth file (.KMZ) of the location of the Arroyo Seco 2 site. (KML)

S2 File Methods. (DOCX)

S3 File Mid-Holocene proyectile points. (DOCX)

S1 Table Map index for Fig 5. (DOCX)

S2 Table The taphonomic modifications identified on extinct Pleistocene mammals. (DOCX)
