# Supplementary figures and images for: The Arrival of Homo sapiens into the Southern Cone at 14,000 Years Ago
Source: PLoS One. 2016 Sep 28;11(9):e0162870. doi: 10.1371/journal.pone.0162870 (PMC5040268; doi:10.1371/journal.pone.0162870)

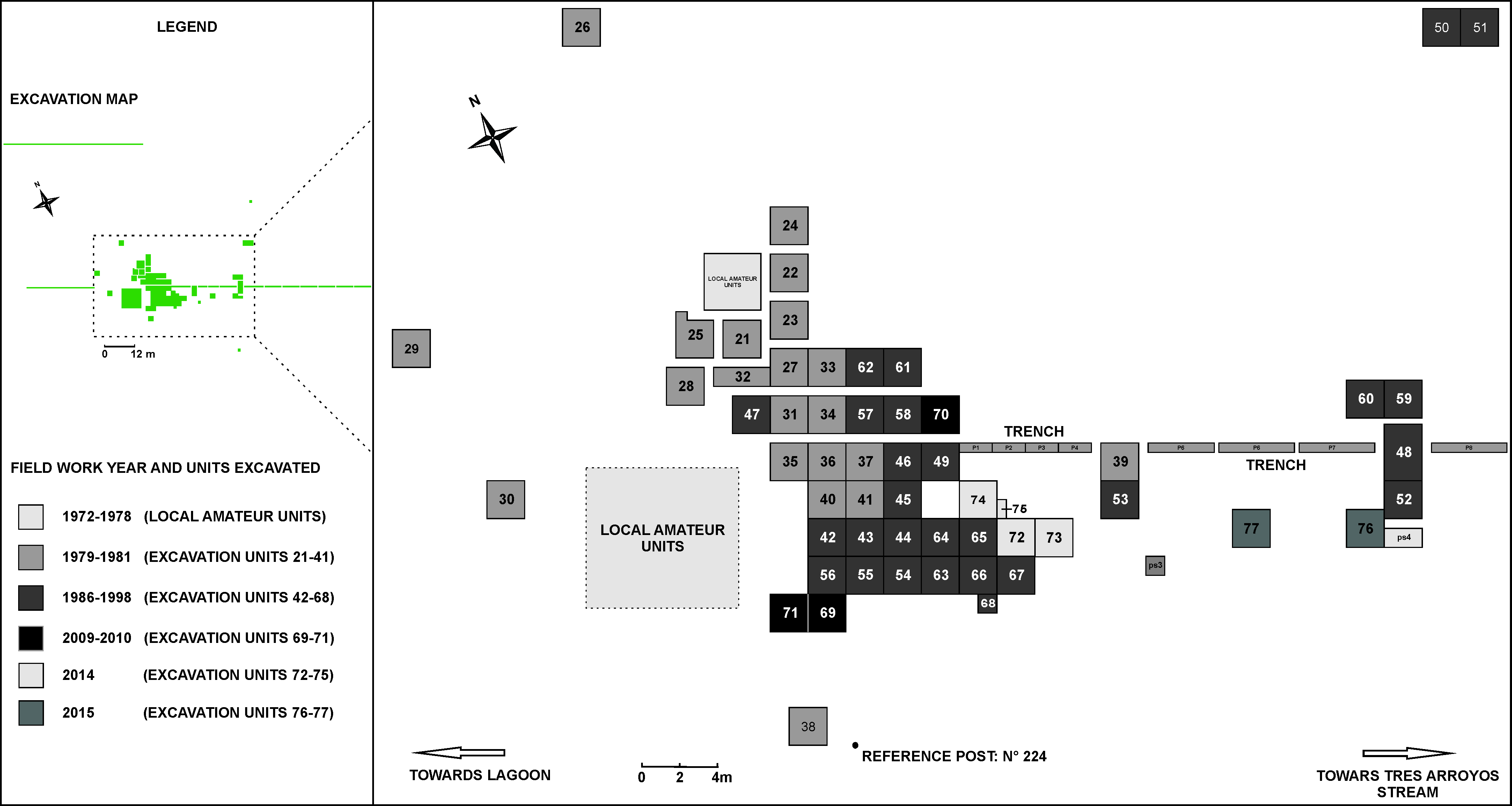

Supplement: S1 Fig — (TIF) [file pone.0162870.s001.tif]

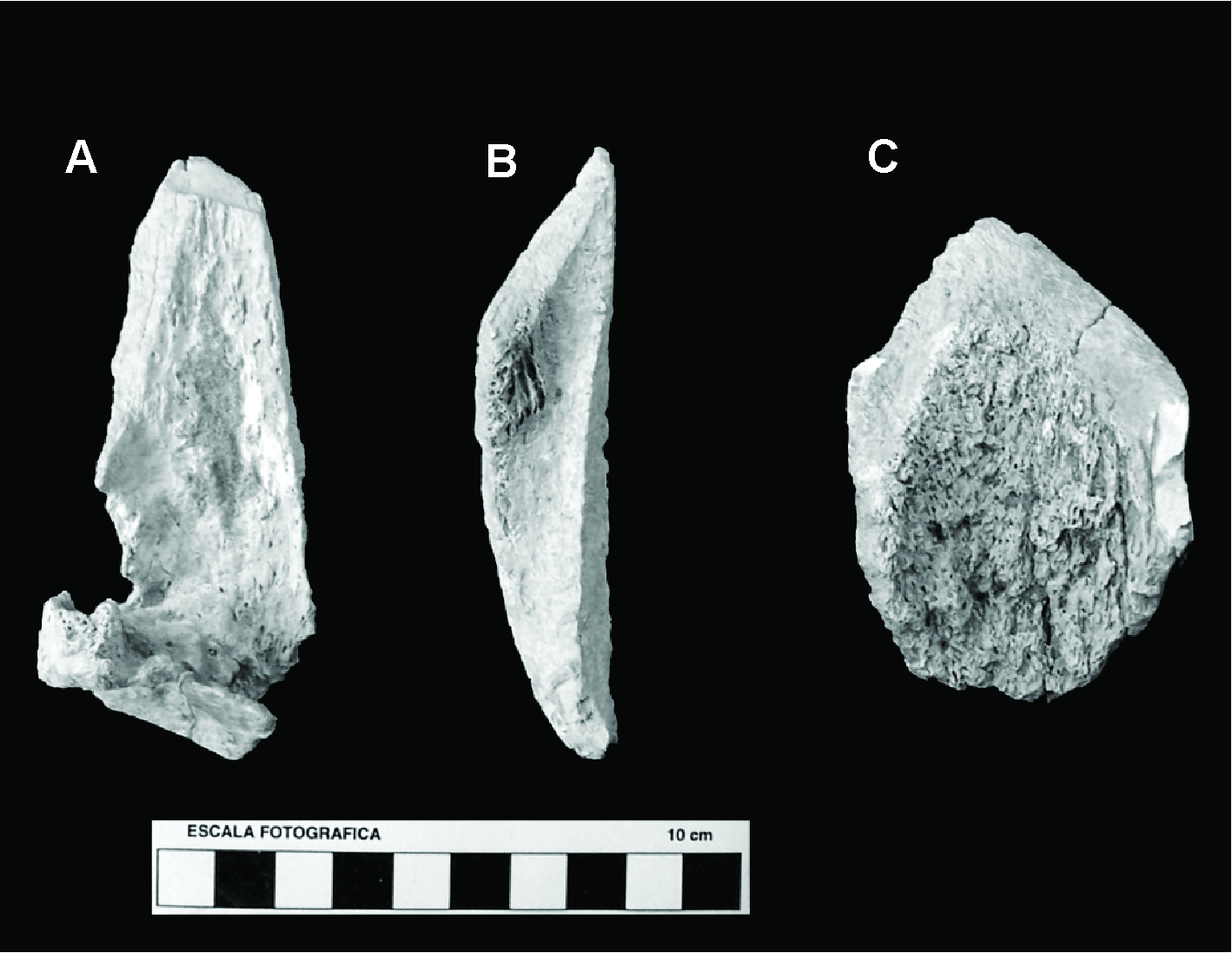

Supplement: S2 Fig — (A-C) indeterminate megamammal fragments (not dated). (TIF) [file pone.0162870.s002.tif]

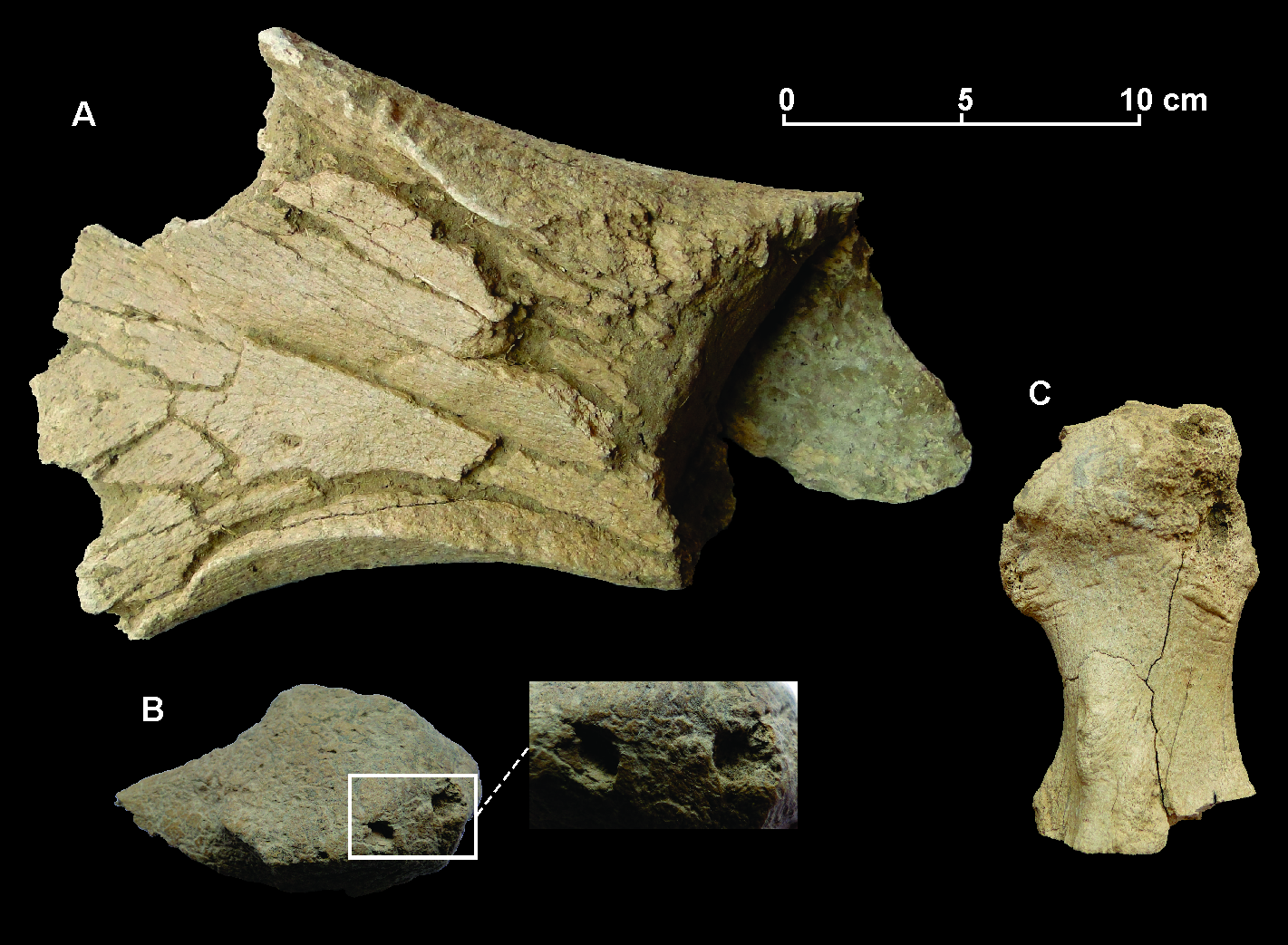

Supplement: S3 Fig — (A) indeterminate megamammal fragment with extensive weathering (not dated). (B) ulna from Equidae sp. with close-up of carnivore punctures (no dated). (C) carpal bone of Toxodon plantensis with weathering and large rodent marks, dated 11,750 ± 70 yr B.P. (CAMS-16389) and 11,590 ± 90 yr B.P. (AA-7964). (TIF) [file pone.0162870.s003.tif]
