# Supplementary material for: The Arrival of Homo sapiens into the Southern Cone at 14,000 Years Ago
Source: PLoS One. 2016 Sep 28;11(9):e0162870. doi: 10.1371/journal.pone.0162870 (PMC5040268; doi:10.1371/journal.pone.0162870)
Supplement: S2 File — (DOCX) [file pone.0162870.s006.docx]

# Methods

The following section provides information on the methods used in the analysis of the different lines of evidence presented in this paper. All specimens involved are publicly deposited in the permanent repository of the Instituto de Investigaciones Arqueológicas y Paleontológicas del Cuaternario Pampeano (INCUAPA-CONICET), Olavarría, Buenos Aires, Argentina; and the Mueso Municipal "José A. Mulazzi" (Municipio de Tres Arroyos), Tres Arroyos, Buenos Aries, Argentina. Specimen numbers are as stated in the text. All aspects and permits for this study are provided under the Direccion Provincial de Patrimonio Cultural del Instituto de Cultura de la Provincia de Buenos Aires (permit number 2014-3-A-125-1).

**Geological sampling**

To define the stratigraphic units at the AS2 site, megascopic identification and sediment samples were taken from the excavation Unit 70 (see Fig 3 in Main Text); focusing on the recognition of color, organic matter content, sedimentary structures, grain size, the presence of biotubation by faunal activity (both invertebrate and vertebrate), and diagenetic carbonate precipitation. In the laboratory, the presence of mechanical sedimentary structures, color, organic material content, granulometry, mineralogical composition, and biological content were ascertained. Dry color was compared using the Munsell color chart. For the granulometric analysis, the corresponding sedimentation unit was processed prior to dispersion by means of ultrasound, with 5% acetic acid, 100 vol. hydrogen peroxide for the elimination of cements and organic material, and with 1% sodium hexametaphosphate as a deflocculant. Particle-size analysis was performed with a Malvern Mastersizer 2000 LASER particle counter. The distribution of particle sizes was measured in damp samples. Particle size distribution was plotted and graphed on a probability paper to process statistically. The different percentile values were used to calculate statistical coefficients according to Folk and Ward (Folk and Ward, 1957). Sediments were classified according to Folk (Folk, 1954). To determine the organic material content, the Walkley and Black (Walkley and Black, 1934) method was employed. Mineralogical composition of very fine sand and coarse silt fractions was carried out by polarization microscopy from loose grain samples prepared with immersion liquid.

**Spatial analysis**

Vertical distribution of extinct Pleistocene fauna and lithic artifacts from the AS2 site were calculated using the total number of tridimensional plotted remains from the excavation units 21-71 (see S1 Fig). The horizontal sample comes from the lower part of stratigraphic Unit Y of the central excavation units 36, 37, 40 to 46.

**Chronology**

Dates expressed in the text are in radiocarbon years before present (^14^C yr B.P.) and calendar years before present (cal yr B.P.) Where calibrated dates are given, these are based on the INTCAL13 calibration curve (Bronk Ramsey, 2009) as implemented in OxCal 4.2 (95.4 confidence interval) age range. The date for *Eutatus seguini* is presented with a 77.3 confidence interval.

**Lithic material**

Lithic technological variability was categorized following the criterion of Aschero (1983, 1975; see Leipus and Landini, 2014). The use of the instruments was determined by microscopic functional analysis following Mansur (1999). For additional information on the functional analysis see Leipus (2014, 2006, 2001) and Leipus and Mansur (2007).

**Faunal Remains**

Taxonomic abundance was quantified using the standard measurements NISP -number of identified specimens- (Payne, 1975) and MNI -minimum numbers of individuals- (White, 1953). Anatomical representation for each taxon was quantified using MNE - minimum number of elements- (Binford, 1984, 1978; Lyman, 1994a, 1994b) and MAU - minimum number of animal units- (Binford, 1984, 1981, 1978).

**Taphonomy**

For each vertebrate remain, the modifications recorded including weathering, sedimentary abrasion, carnivore marks, rodent marks, root etching, manganese stains, thermal alteration, calcium carbonate, chemical deterioration and trampling were determined following the methods described in Behrensmeyer (1978), Binford (1981), Grayson (1984), Gutiérrez and Kaufmann (2007), Haynes (1980), Lyman (1994b); Olsen and Shipman (1988), Shipman (1981); among others. Anthropic modification attributes recorded including cut marks, thermal alteration, and bone fractures were determined following the methods described in Binford (1981), Blumenshine and Selvaggio (1988); Blumenshine et al. (1996), Domínguez-Rodrigo et al. (2009), Fisher (1995), Galán et al. (2009), Johnson (1985), Lyman (1994b), Olsen and Shipman (1988), among others.

# References

Aschero, C.A., 1975. Ensayo para una clasificación morfológica de artefactos líticos. Informe CONICET.

Aschero, C.A., 1983. Registro de códigos para atributos descriptivos aplicados a artefactos líticos. Informe CONICET.

Behrensmeyer, A.K., 1978. Taphonomic and ecologic information from bone weathering. Paleobiology 4, 150–162. doi:10.2307/2400283

Binford, L.R., 1978. Nunamiut Ethnoarchaeology. Academic Press, New York, United States.

Binford, L.R., 1981. Bones: ancient men and modern myths. Academic Press, New York, United States.

Binford, L.R., 1984. Faunal remains from Klasies River Mouth, Studies in Archaeology. Academic Press, Orlando.

Blumenschine, R.J., Marean, C.W., Capaldo, S.D., 1996. Blind tests of inter-analyst correspondence and accuracy in the identification of cut marks, percussion marks and carnivore tooth marks on bone surfaces. Journal of Archaeological Science 23, 493–507.

Blumenschine, R.J., Selvaggio, M.M., 1988. Percussion marks on bone surfaces as a new diagnostic of hominid behaviour. Nature 333, 763–765. doi:10.1038/332141a0

Bronk Ramsey, C., 2009. Bayesian analysis of radiocarbon dates. Radiocarbon 51, 337–360.

Domínguez-Rodrigo, M., de Juana, S., Galán, A.B., Rodríguez, M., 2009. A new protocol to differentiate trampling marks from butchery cut marks. Journal of Archaeological Science 36, 2643–2654. doi:10.1016/j.jas.2009.07.017

Fisher Jr., J.W., 1995. Bone Surface Modifications in Zooarchaeology. Journal of Archaeological Method and Theory 2, 7–68.

Folk, R.L., 1954. The distinction between grain size and mineral composition in sedimentary rock nomenclature. Journal of Geology 62, 344–359.

Folk, R.L., Ward, C.L., 1957. The Brazos fiver bar: a study on the significance of grain-size parameters. Journal of Sedimentary Petrology 27, 3–27.

Galán, A.B.B., Rodríguez, M., de Juana, S., Domínguez-Rodrigo, M., 2009. A new experimental study on percussion marks and notches and their bearing on the interpretation of hammerstone-broken faunal assemblages. Journal of Archaeological Science 36, 776–784. doi:10.1016/j.jas.2008.11.003

Grayson, D.K., 1984. Quantitative Zooarchaeology: Topics in the Analysis of Archaeological Faunas. Academic Press, Orlando.

Gutiérrez, M.A., Kaufmann, C.A., 2007. Criteria for the identification of formation processes in guanaco (Lama guanicoe) bone assemblages in fluvial-lacustrine environments. Journal of Taphonomy 5, 151–175.

Haynes, G., 1980. Evidence of carniore gnawing on Pleistocene and Recent mamalian bones. Paleobiology 6, 341–351.

Johnson, E., 1985. Current Developments in Bone Technology., in: Schiffer, M.B. (Ed.), Advances in Archaeological Method and Theory, Vol. 8. New York: Academic Press, pp. 157–235.

Leipus, M.S., 2001. Análisis de rastros de uso experimentales en materias primas líticias de la Región Pampeana. Arqueología Uruguaya hacia Fines del Milenio I, 491–503.

Leipus, M.S., 2006. Análisis de los modos de uso prehispánicos de las materias primas líticas en el Sudeste de la región Pampeana: Una aproximación fucional. Universidad Nacional de La Plata, La Plata, Argentina.

Leipus, M.S., 2014. Análisis funcional de base microscópica de los instrumentos líticos manufacturados por talla de las unidades estratigráficas Y, S y Z, in: Politis, G.G., Gutiérrez, M.A., Scabuzzo, C. (Eds.), Estado Actual de Las Investigaciones En El Sitio Arqueológico Arroyo Seco 2 (Partido de Tres Arroyos, Provincia de Buenos Aires, Argentina). Universidad Nacional del Centro de la Provincia de Buenos Aires. Facultad de Ciencias Sociales, Olavarría, Argentina, pp. 229–274.

Leipus, M.S., Landini, M.C., 2014. Materias primas y tecnología: un estudio compartivo del material lítico, in: Politis, G.G., Gutiérrez, M.A., Scabuzzo, C. (Eds.), Estado Actual de Las Investigaciones En El Sitio Arqueológico Arroyo Seco 2 (Partido de Tres Arroyos, Provincia de Buenos Aires, Argentina). Universidad Nacional del Centro de la Provincia de Buenos Aires. Facultad de Ciencias Sociales, Olavarría, Argentina, pp. 179–228.

Leipus, M.S., Mansur, M.E., 2007. El análisis funcional de base microscópica aplicado a materiales heterogéneos. Persepectivas metodológicas para el estudio de as cuarcitas pampeanas., in: Bayón, C., Flegenheimer, N., González de Bonaveri, M.I., Frére, M. (Eds.), Arqueología En Las Pampas. Sociedad Argentina de Antropología, Bahía Blanca, pp. 179–200.

Lyman, R.L., 1994a. Quantitative Units and Terminology in Zooarchaeology. American Archaeology 59, 36–71.

Lyman, R.L., 1994b. Vertebrate Taphonomy. Cambridge University Press, Cambridge.

Mansur, M.E., 1999. Análisis Funcional de instrumentos líticos, in: Actas Del XII Congreso Nacional de Arqueología. La Plata, pp. 355–366.

Olsen, S.L., Shipman, P., 1988. Surface modification on bone: Trampling versus butchery. Journal of Archaeological Science 15, 535–553. doi:10.1016/0305-4403(88)90081-7

Payne, S., 1975. Partial recovery and sample bias, in: Clason, A.T. (Ed.), Archaezoological Studies. American Elsevier, New York, pp. 7–17.

Shipman, P., 1981. Life History of a Fossil. An Introduction to Taphonomy and Paleoecology. Harvard University Press, Cambridge.

Walkley, A., Black, I.A., 1934. A Examination of a Rapid Methord for determination of organic carbon in sois-effect of variation in Digestion conditions and Inorganic soil constituents. Soil Science 63, 251–257.

White, T.D., 1953. A method of calculating the dietary percentage of various food animals utilized by aboriginal peoples. American Antiquity 19, 396–398.
