# Supplementary material for: The Arrival of Homo sapiens into the Southern Cone at 14,000 Years Ago
Source: PLoS One. 2016 Sep 28;11(9):e0162870. doi: 10.1371/journal.pone.0162870 (PMC5040268; doi:10.1371/journal.pone.0162870)
Supplement: S3 File — (DOCX) [file pone.0162870.s007.docx]

# Mid-Holocene Projectile Points

In total, 18 projectile points recovered from the site. Eleven of the projectile points were recovered between the bone elements of four human skeletons (dated in ca. 7800 to 7600 ^14^C yr B.P). The seven projectile points not associated with human remains are bifacial and appendicular triangular shaped; two of these points come from the upper levels of Unit X and five from upper levels of Unit Y. Morphological analysis of the projectile points, both associated and not associated with the human skeletons appear to have entered the site at a final production stage or in a state of advanced manufacturing (Escola, 2014). There is no chronological overlap between the oldest projectile points of the site (those lodged in the skeletons) and the dates of the extinct Pleistocene fauna.

# References

Escola, P., 2014. Proyectiles líticos en contexto en Arroyo Seco 2: algo más que una tecnología para la caza, in: Politis, G.G., Gutiérrez, M.A., Scabuzzo, C. (Eds.), Estado Actual de Las Investigaciones En El Sitio Arqueológico Arroyo Seco 2 (Partido de Tres Arroyos, Provincia de Buenos Aires, Argentina). Universidad Nacional del Centro de la Provincia de Buenos Aires, Olavarría, Argentina, pp. 313–328.
