# Supplementary material for: The Arrival of Homo sapiens into the Southern Cone at 14,000 Years Ago
Source: PLoS One. 2016 Sep 28;11(9):e0162870. doi: 10.1371/journal.pone.0162870 (PMC5040268; doi:10.1371/journal.pone.0162870)
Supplement: S1 Table — (DOCX) [file pone.0162870.s008.docx]

**S1 Table. Map index for Fig 5.**

| **Taxa / Map Number** | **Element** | **Bone portion** | **Excavation Unit** | **Storage ID Number** |
| --- | --- | --- | --- | --- |
| ***Equus neogeus*** |  |  |  |  |
| 6 | 3rd right phalanx | complete | 40 | AS2.40.13 |
| 7 | phalanx | distal epiphysis | 42 | FCS.AS2.10334 |
| 8 | metatarsal | shaft and proximal epiphysis | 43 | AS2.43.211 |
| 9 | metatarsal | proximal epiphysis | 45 | AS2.45.16 |
| 10 | left humerus | distal epiphysis | 41 | AS2.41.34 |
| 11 | metapodial | distal epiphysis | 37 | AS2.37.20 |
|  |  |  |  |  |
| ***Hippidion* sp.** |  |  |  |  |
| 14 | 2nd phalanx | complete | 45 | AS2.45.17 |
|  |  |  |  |  |
| ***Macrauchenia patachonica*** | |  |  |  |
| 12 | left tibia | distal epiphysis | 46 | AS2.46.6 |
|  |  |  |  |  |
| ***Magetherium americanum*** | |  |  |  |
| 1 | left tibia | distal shaft | 36 | AS2.36.9 |
| 2 | right tibia | shaft | 36 | AS2.36.10 |
| 3 | left tibia | proximal shaft | 41 | FCS.AS2.8487 |
| 4 | right femur | shaft | 36 | AS2.36.11 |
| 5 | left femur | shaft | 40 | AS2.40.8 |
|  |  |  |  |  |
| ***Mylodon* sp. (*Mylodontinae*)** | |  |  |  |
| 38 | dermal bone | complete | 37 | FCS.AS2.6353 |
| 78 | dermal bone | complete | 45 | FCS.AS2.8479 |
| 82 | dermal bone | complete | 44 | FCS.AS2.10363 |
| 83 | dermal bone | complete | 40 | FCS.AS2.10365 |
| 85 | dermal bone | complete | 45 | FCS.AS2.10370 |
|  |  |  |  |  |
| ***Camelidae* cf *Hemiauchenia*** | |  |  |  |
| 13 | 2nd right molar | semi-complete | 40 | FCS.AS2.10351 |
|  |  |  |  |  |
| **Megamammal sp.** |  |  |  |  |
| 33 | long bone | shaft | 37 | FCS.AS2.6337 |
| 34 | mandible | body | 37 | FCS.AS2.6347 |
| 39 | cancellous bone | fragment | 37 | FCS.AS2.6366 |
| 44 | compact bone | fragment | 40 | FCS.AS2.6657 |
| 69 | compact bone | fragment | 44 | FCS.AS2.7860 |
| 79 | cancellous bone | fragment | 41 | FCS.AS2.10236 |
| 81 | radius | proximal epiphysis | 43 | FCS.AS2.10355 |
| 84 | long bone | shaft | 45 | FCS.AS2.10369 |
| 87 | fragment |  | 45 | FCS.AS2.10374 |
| 93 | long bone | shaft | 43 | FCS.AS2.10230 |
| 98 | long bone | shaft | 36 | FCS.AS2.16126 |
| 99 | long bone | shaft | 36 | FCS.AS2.16127 |
|  |  |  |  |  |
| ***Lama guanicoe*** |  |  |  |  |
| 31 | right cuneiform | complete | 36 | FCS.AS2.6275 |
| 35 | left tibia | distal epiphysis | 37 | FCS.AS2.6349 |
| 36 | lumbar vertebrae | right anterior joint | 37 | FCS.AS2.6351 |
| 40 | phalanx | shaft and proximal epiphysis | 37 | FCS.AS2.6369 |
| 41 | right humerus | shaft | 37 | FCS.AS2.6373 |
| 42 | skull | tympanic bone | 37 | FCS.AS2.6378 |
| 43 | right calcaneus | tuberosity and body | 37 | FCS.AS2.6379 |
| 45 | right astragalus | complete | 40 | FCS.AS2.6659 |
| 46 | cervical vertebrae | body cap | 40 | FCS.AS2.6661 |
| 47 | bezoar | complete | 40 | FCS.AS2.6663 |
| 51 | 2nd phalanx | proximal epiphysis | 41 | FCS.AS2.6769 |
| 53 | left tibia | distal epiphysis | 41 | FCS.AS2.6773 |
| 54 | left cuneiform | complete | 41 | FCS.AS2.6774 |

**S1 Table. Cont.**

| **Taxa / Map Number** | **Element** | **Bone portion** | **Excavation Unit** | **Storage ID Number** |
| --- | --- | --- | --- | --- |
| ***Lama guanicoe*** |  |  |  |  |
| 55 | left calcaneus | semi-complete | 41 | FCS.AS2.6775 |
| 57 | 2nd phalanx | proximal epiphysis | 42 | FCS.AS2.6947 |
| 58 | left radius-ulna | carpal radius | 42 | FCS.AS2.6950 |
| 61 | left scaphoid | complete | 46 | FCS.AS2.7136 |
| 62 | pre-molar | semi-complete | 43 | FCS.AS2.7742 |
| 63 | left scaphoid | complete | 43 | FCS.AS2.7745 |
| 64 | mandible | 4th premolar cavity | 43 | FCS.AS2.7747 |
| 65 | left radius-ulna | proximal epiphysis | 43 | FCS.AS2.7749 |
| 67 | atlas | ventral arc | 43 | FCS.AS2.7753 |
| 68 | 1st phalanx | distal epiphysis | 43 | FCS.AS2.7754 |
| 70 | cervical vertebrae | semi-complete | 44 | FCS.AS2.7861 |
| 71 | right unciform | complete | 44 | FCS.AS2.7862 |
| 72 | skull | left tympanic bone | 44 | FCS.AS2.7863 |
| 73 | left trapezoid | complete | 44 | FCS.AS2.7866 |
| 74 | left cuboid | complete | 44 | FCS.AS2.7867 |
| 76 | right fibula | complete | 45 | FCS.AS2.7971 |
| 77 | right unciform | complete | 45 | FCS.AS2.7972 |
| 86 | scaphoid | semi-complete | 45 | FCS.AS2.10373 |
| 88 | left tibia | articular surface of the talus | 42 | FCS.AS2.14117 |
| 95 | left femur | proximal epiphysis | 36 | AS2.36.3 |
|  |  |  |  |  |
| ***Ozotoceros bezoarticus*** | |  |  |  |
| 32 | 1st phalanx | complete | 36 | FCS.AS2.6276 |
| 37 | molar | complete | 37 | FCS.AS2.6352 |
| 50 | left metatarsal | proximal epiphysis | 41 | FCS.AS2.6766 |
| 52 | carpal bone | semi-complete | 41 | FCS.AS2.6771 |
| 59 | right tibia | proximal epiphysis | 42 | FCS.AS2.6951 |
| 60 | patella | semi-complete | 46 | FCS.AS2.7135 |
|  |  |  |  |  |
| ***Rhea americana*** |  |  |  |  |
| 56 | 5th phalanx | complete | 41 | FCS.AS2.6778 |
| 75 | 5th phalanx | complete | 45 | FCS.AS2.7969 |
|  |  |  |  |  |
| ***Dolichotis patagonum*** |  |  |  |  |
| 49 | humerus | distal epiphysis | 41 | FCS.AS2.6762 |
|  |  |  |  |  |
| ***Chaetophractus villosus*** | |  |  |  |
| 48 | skull | fragment | 41 | FCS.AS2.6761 |
|  |  |  |  |  |
| ***Lagostomus maximus*** |  |  |  |  |
| 66 | calcaneus | complete | 43 | FCS.AS2.7750 |
|  |  |  |  |  |
| **Mammal sp.** |  |  |  |  |
| 15 | fragment |  | 37 | FCS.AS2.16128 |
| 30 | long bone | epiphysis | 36 | FCS.AS2.6274 |
| 80 | long bone | shaft | 41 | FCS.AS2.10237 |
| 89 | rib | shaft | 42 | FCS.AS2.14132 |
|  |  |  |  |  |
| **indeterminate** |  |  |  |  |
| 17 | fragment |  | 44 | AS2.44.15 |
| 18 | fragment |  | 41 |  |
|  |  |  |  |  |
| **Lithic artifacts** |  |  |  |  |
| 16 | bilateral retouched edge tool | | 41 | AS2.41.25 |
| 19 | double side scraper (quartzite) | | 41 |  |
| 20 | scraper with front edge perimeter (quartzite) | | 41 | AS2.41.31 |
| 21 | front edge scraper (quartzite) | | 46 | AS2.46.6 |
| 22 | rounded oval shaped cobble (rhyolite) | | 40 | AS2.41.30 |
| 23 | rounded oval shaped cobble (lutita) | | 37 | AS2.37.8 |
| 24 | medium sized flake (quartzite) | | 45 |  |
| 25 | medium sized flake (quartzite) | | 45 |  |
| 26 | medium sized flake (quartzite) | | 45 |  |
| 27 | medium sized flake (quartzite) | | 45 |  |
| 28 | medium sized flake (quartzite) | | 45 |  |
| 29 | medium sized flake (chalcedony) | | 36 | AS2.36.21 |

**S1 Table. Cont.**

| **Taxa / Map Number** | **Element** | **Bone portion** | **Excavation Unit** | **Storage ID Number** |
| --- | --- | --- | --- | --- |
|  |  |  |  |  |
| **Lithic artifacts** |  |  |  |  |
| 90 | flake |  | 41 |  |
| 91 | flake |  | 44 |  |
| 92 | flake |  | 44 |  |
| 94 | retouched edge tool (quartzite) | | 41 | AS2.41.29 |
| 96 | double convergent blunt scraper (quartzite) | | 36 | AS2.36.18 |
| 97 | front edge retouched notch | | 37 | AS2.37.7 |
| 100 | large sized flake (quartzite) | | 46 |  |
