# Supplementary material for: The Arrival of Homo sapiens into the Southern Cone at 14,000 Years Ago
Source: PLoS One. 2016 Sep 28;11(9):e0162870. doi: 10.1371/journal.pone.0162870 (PMC5040268; doi:10.1371/journal.pone.0162870)
Supplement: S2 Table — (DOCX) [file pone.0162870.s009.docx]

**S2 Table. The taphonomic modifications identified on extinct Pleistocene mammals**

| Taxon | S |  | CB | |  | DF | |  | FF | |  | W | |  | CC | |
| --- | --- | --- | --- | --- | --- | --- | --- | --- | --- | --- | --- | --- | --- | --- | --- | --- |
|  | N |  | N | % |  | N | % |  | N | % |  | N | % |  | N | % |
| *Equus neogeus* | 8 |  | 1 | 12.5 |  | 4 | 50.0 |  | 3 | 37.5 |  | 3 | 37.5 |  | 2 | 25.0 |
| *Eutatus seguini* | 2 |  | 0 | 0.0 |  | 2 | 100 |  | 0 | 0.0 |  | 1 | 50.0 |  | 2 | 100 |
| *Glossotherium robustum* | 2 |  | 0 | 0.0 |  | 2 | 100 |  | 0 | 0.0 |  | 2 | 100 |  | 1 | 50.0 |
| *Megatherium americanum* | 2 |  | 0 | 0.0 |  | 2 | 100 |  | 0 | 0.0 |  | 2 | 100 |  | 2 | 100 |
| *Toxodon platensis* | 1 |  | 0 | 0.0 |  | 1 | 100 |  | 0 | 0.0 |  | 1 | 100 |  | 1 | 100 |
| *Glypotodon* sp. | 1 |  | 0 | 0.0 |  | 1 | 100 |  | 0 | 0.0 |  | 1 | 100 |  | 0 | 0.0 |
| *Hippidion* sp. | 3 |  | 1 | 33.0 |  | 2 | 67.0 |  | 0 | 0.0 |  | 2 | 67.0 |  | 2 | 67.0 |
| *Macrauchenia* sp. | 3 |  | 3 | 100 |  | 0 | 0.0 |  | 0 | 0.0 |  | 3 | 100 |  | 3 | 100 |
| *Mylodon* sp. | 0 |  | 0 | 0.0 |  | 0 | 0.0 |  | 0 | 0.0 |  | 0 | 0.0 |  | 0 | 0.0 |
| Camelidae cf. Hemiauchenia | 9 |  | 4 | 11.0 |  | 5 | 55.5 |  | 0 | 0.0 |  | 1 | 11.0 |  | 3 | 33.0 |
| Lestodontinae cf. *Lestondon* | 1 |  | 0 | 0.0 |  | 1 | 100 |  | 0 | 0.0 |  | 1 | 100 |  | 1 | 100 |
| Equidae | 16 |  | 0 | 0.0 |  | 16 | 100 |  | 0 | 0.0 |  | 14 | 87.5 |  | 10 | 62.5 |
| Megamammal sp. | 116 |  | 0 | 0.0 |  | 100 | 86.0 |  | 6 | 5.0 |  | 58 | 50.0 |  | 57 | 49.0 |
| Total | 164 |  | 9 | 5.5 |  | 136 | 83.0 |  | 9 | 5.5 |  | 89 | 54.0 |  | 84 | 51.0 |

**S2 Table. Cont.**

| Taxon | CD | |  | RM | |  | RoM | |  | M | |  | GA | |  | CM | |  | TA | |
| --- | --- | --- | --- | --- | --- | --- | --- | --- | --- | --- | --- | --- | --- | --- | --- | --- | --- | --- | --- | --- |
|  | N | % |  | N | % |  | N | % |  | N | % |  | N | % |  | N | % |  | N | % |
| *Equus neogeus* | 8 | 100 |  | 2 | 25.0 |  | 2 | 25.0 |  | 3 | 37.5 |  | 1 | 12.5 |  | 0 | 0.0 |  | 0 | 0.0 |
| *Eutatus seguini* | 0 | 0.0 |  | 2 | 100 |  | 2 | 100 |  | 0 | 0.0 |  | 0 | 0.0 |  | 0 | 0.0 |  | 0 | 0.0 |
| *Glossotherium robustum* | 2 | 100 |  | 1 | 50.0 |  | 0 | 0.0 |  | 2 | 100 |  | 0 | 0.0 |  | 0 | 0.0 |  | 0 | 0.0 |
| *Megatherium americanum* | 2 | 100 |  | 1 | 50.0 |  | 1 | 50.0 |  | 1 | 50.0 |  | 0 | 0.0 |  | 0 | 0.0 |  | 0 | 0.0 |
| *Toxodon platensis* | 1 | 100 |  | 1 | 100 |  | 1 | 100 |  | 1 | 100 |  | 0 | 0.0 |  | 0 | 0.0 |  | 0 | 0.0 |
| *Glypotodon* sp. | 1 | 100 |  | 1 | 100 |  | 1 | 100 |  | 0 | 0.0 |  | 0 | 0.0 |  | 0 | 0.0 |  | 0 | 0.0 |
| *Hippidion* sp. | 2 | 67.0 |  | 1 | 33.0 |  | 1 | 33.0 |  | 1 | 33.0 |  | 0 | 0.0 |  | 0 | 0.0 |  | 0 | 0.0 |
| *Macrauchenia* sp. | 0 | 0.0 |  | 2 | 67.0 |  | 2 | 67.0 |  | 2 | 67.0 |  | 0 | 0.0 |  | 0 | 0.0 |  | 0 | 0.0 |
| *Mylodon* sp. | 0 | 0.0 |  | 0 | 0.0 |  | 0 | 0.0 |  | 0 | 0.0 |  | 0 | 0.0 |  | 0 | 0.0 |  | 0 | 0.0 |
| Camelidae cf. Hemiauchenia | 2 | 22.0 |  | 5 | 56.0 |  | 5 | 56.0 |  | 3 | 33.0 |  | 0 | 0.0 |  | 0 | 0.0 |  | 0 | 0.0 |
| Lestodontinae cf. *Lestondon* | 0 | 0.0 |  | 1 | 100 |  | 1 | 100 |  | 1 | 100 |  | 0 | 0.0 |  | 0 | 0.0 |  | 0 | 0.0 |
| Equidae | 12 | 75.0 |  | 11 | 69.0 |  | 11 | 69.0 |  | 7 | 44.0 |  | 0 | 0.0 |  | 2 | 12.5 |  | 0 | 0.0 |
| Megamammal sp. | 46 | 38.0 |  | 38 | 33.0 |  | 31 | 27.0 |  | 30 | 26.0 |  | 4 | 3.0 |  | 0 | 0.0 |  | 1 | 1.0 |
| Total | 76 | 46.0 |  | 66 | 40.0 |  | 58 | 35.0 |  | 51 | 31.0 |  | 5 | 3.0 |  | 2 | 1.0 |  | 1 | 0.5 |

Taphonomic modifications are abbreviated as follows: S, Sample Analyzed; CB, Complete Bone; DF, Dry Fracture; FF, Fresh Fracture; W, Weathering; CC, Calcium Carbonate (CaC03); CD, Chemical Deterioration; RM, Root Marks; RoM, Rodent Marks; M, Manganese; GA, Geological Abrasion; CM, Carnivore Marks; TA, Thermal Alteration; N, number of specimens.
